# CB_1_ receptors on a subset of vagal afferent neurons modulate voluntary ethanol intake in mice

**DOI:** 10.1038/s41380-025-03266-9

**Published:** 2025-09-20

**Authors:** Alexa Herrerias, Anna Oliverio, Szabolcs Dvorácskó, Arthi Thyagarajan, Lee Chedester, Jie Liu, Resat Cinar, Malliga R. Iyer, George Kunos, Grzegorz Godlewski

**Affiliations:** 1 Laboratory of Physiologic Studies, Division of Intramural Clinical and Biological Research, National Institute on Alcohol Abuse and Alcoholism, National Institutes of Health (NIH), Bethesda, MD 20892 USA; 2 Section on Fibrotic Disorders, Division of Intramural Clinical and Biological Research, National Institute on Alcohol Abuse and Alcoholism, National Institutes of Health (NIH), Bethesda, MD 20892 USA; 3 Section on Medicinal Chemistry, Division of Intramural Clinical and Biological Research, National Institute on Alcohol Abuse and Alcoholism, National Institutes of Health (NIH), Bethesda, MD 20892 USA; 4 Laboratory of Biomolecular Structure and Pharmacology, Institute of Biochemistry, HUN-REN Biological Research Centre, Szeged, Hungary

**Keywords:** Neuroscience, Physiology, Drug discovery, Psychology

## Abstract

Gut-brain signaling influences alcohol consumption and addiction behaviors. We found that selectively deleting cannabinoid receptor 1 (CB_1_R) from advillin^+^ peripheral sensory neurons eliminates the inhibitory effect of the peripheral CB_1_R antagonist JD5037 on voluntary ethanol intake (VEI). Similar results were seen in mice with CB_1_R deletion in Phox2b^+^ nodose ganglia (NGA), but not in Wnt^+^ dorsal root ganglia. These findings were corroborated with MRI-1891, another non-brain penetrant CB_1_R antagonist. The inhibition of VEI by JD5037 was lost in *Gpr65*^Cre^;*Cnr1*^*lox/lox*^ mice but remained intact in Glp1r^Cre^;*Cnr1*^*lox/lox*^ mice. Additionally, deleting the ghrelin receptor (Ghsr) from Phox2b^+^ NGA neurons blocked the inhibition of alcohol intake either by a Ghsr or by CB_1_R antagonists. Thus, CB_1_R on Gpr65^+^ NGA projections to the mucosa of the gastrointestinal tract is essential for VEI. These findings also suggest a mutual interdependence of endocannabinoid and ghrelin signaling in controlling VEI via a gut-brain axis.

## Introduction

The preference for drugs, palatable foods, and beverages is rooted in their ability to activate brain reward pathways [1]. Compounds like ethanol, nicotine, and Δ^9^-tetrahydrocannabinol (THC), the psychoactive component of marijuana that interacts with receptors for endogenous cannabinoids (ECs), act directly on the mesolimbic dopamine circuits that elicit hedonic desire [2]. Specific dopaminergic neurons also monitor gustatory information and hunger signals originating from specialized gut sensor cells and peripheral neurons that are, among others, influenced by ECs [3–9]. Consequently, targeted inhibition of vagal endocannabinoid tone has been shown to reduce the “munchies” (i.e., palatable eating) in a vagus nerve-dependent manner [10, 11]. Alcohol is unique in a way that in addition to its direct addictive properties, it elicits sensory experiences and drives energy intake. These characteristics contribute to its widespread use and pose a challenge for treating alcohol use disorder (AUD). Available pharmaceuticals are only effective in a subset of individuals [12].

There is a growing recognition of the cannabinoid receptor-1 (CB_1_R) as an important regulator of voluntary alcohol intake [13] (VEI). Much of the work focused on the interaction between the endocannabinoid system (ECS) and alcohol in the ventral tegmental area, nucleus accumbens (NAcc), and prefrontal cortex, where CB_1_R is highly expressed [14]. Studies demonstrated that genetic deletion or pharmacological inhibition of CB_1_R in these locations reduces VEI and decreases ethanol-induced spikes in dopamine release in the NAcc, whereas CB_1_R agonists increase ethanol intake [15, 16]. The importance of peripheral CB_1_R in regulating ethanol preference was suggested by the fact that both the brain-penetrant CB_1_R antagonist rimonabant and its non-brain-penetrant counterpart JD5037 inhibited ethanol intake upon systemic, but not intracerebroventricular, administration [17]. Additionally, the gut-brain axis has been recognized as a potential regulator of VEI, with peptides like ghrelin being of particular interest [18]. Antagonists of the ghrelin receptor (Ghsr) suppressed ethanol consumption and its rewarding properties [19]. ECs have recently been proposed to facilitate ghrelin acylation via CB_1_R in ghrelin-producing cells and promote alcohol intake in a vagus nerve-dependent manner [20]. These findings underscore the role of peripheral CB_1_R, Ghsr, and the gut-brain axis in alcohol intake, highlighting their potential as therapeutic targets for AUD.

Current single-cell RNA-sequencing data and other histological studies demonstrated the existence of distinct subsets of peripheral sensory nerve terminals of vagal and spinal afferents that innervate the gastrointestinal (GI) tract, each identified by specific genetic markers and functions [9, 21–26]. These studies also indicated the presence of CB_1_R-positive and/or Ghsr-positive neurons in nodose ganglia (NGA) and dorsal root ganglia (DRG). It remains unclear which population of sensory afferent neurons is engaged in CB_1_R-mediated control of VEI. This prompted us to test alcohol drinking behavior in transgenic mouse lines lacking CB_1_R in different subpopulations of neurons of the gut-brain axis. We show that selective deletion of CB_1_R in nodose ganglia, specifically from the Gpr65^+^ subpopulation projecting to the mucosal layer of the GI tract, is essential to occlude the inhibitory effect of peripheral CB_1_R antagonists on VEI. The effect of drugs appears only when Ghsr is present in NGA neurons.

## Materials and methods

### Ethics approval and consent to participate

All animal methods were performed in accordance with the ethical guidelines for animal research established by the US government regulations, including the Animal Welfare Act. Our animal study protocol (registration number: LPS-GK-1) was reviewed and approved by the Institutional Animal Care and Use Committee (IACUC) at the National Institute of Alcohol Abuse and Alcoholism (NIAAA) of the National Institutes of Health (NIH). This approval was based on the certification of several NIAAA/NIH IACUC board members and officials: Facility Veterinarian (certified animal welfare standards), Facility Manager (confirmed resource capabilities within the animal facility), Safety Representatives (reviewed and concurred with the use of any hazardous agents or radioactive materials), Scientific Director of NIAAA (approved the protocol based on scientific merit, rigor, and reproducibility, including the consideration of sex as a biological variable). The final certification and approval note were provided by the Chairperson of the NIAAA/NIH IACUC.

### Animals

Mice were housed 2–4 animals per cage in air-conditioned racks maintained at 22.0 ± 0.5 °C and 50.0 ± 1.0% humidity, under a 12:12 light/dark cycle. They had *ad libitum* access to food (NIH-31 standard rodent chow) and water. Mice used in drinking paradigms were 10 ± 2 weeks old and weighing 22–26 g (males) and 17–21 g (females) at the onset of the study. Details regarding strains used in the study and *Ghsr*^*lox/lox*^ development and are available in the Supplementary Material.

### Genotyping

Genotyping was performed from tail or ear clippings using a REDExtract-N-Amp^TM^ tissue PCR kit (MilliporeSigma; USA) according to the manufacturer’s protocol. Oligonucleotides for genotyping were from ThermoFisher Scientific (USA). Sequences and product sizes are provided in the Supplementary Material. PCR products were separated on 3% UltraPure^TM^ Agarose gel (Thermo Fisher Scientific; USA) and visualized on G:BOX scanner (Syngene, USA).

### Fluorescent in situ hybridization

Fresh-frozen mouse nodose ganglion and dorsal root ganglion sections (14 µm) and paraffin-embedded stomach tissue sections (4 μm) were prepared and processed for fluorescent in situ hybridization [27] (FISH) with the RNAscope® Multiplex Fluorescent Kit v2 (Advanced Cell Diagnostics; USA) according to the manufacturer’s instructions and analyzed using a Zeiss LSM700 (USA) confocal microscope. RNAscope^®^ FISH probes and fluorophores are listed in the Supplementary Material.

### Two-bottle choice test

The test was performed as described [28]. Animals had continuous access to water and 15% ethanol for 10 days. Starting on day 5, mice received vehicle (control group) by oral gavage or JD5037 (3 mg/kg), MRI-1891 (1 mg/kg or 3 mg/kg) or PF-5190457 (3 mg/kg). Mice were randomized based on their phenotypic features, e.g., body weights, drinking and eating habits. The treatment regimen continued for 5 days, always one hour before the dark period. Animals were euthanized for blood collection 12–16 h after the last treatment day and processed as described before [20].

### Drinking in the dark paradigm

The procedure was performed as described before [29]. Mice had limited access to 20% ethanol for 4 h starting three hours into the dark cycle. The drinking pattern was monitored for 3 days. One hour before the dark period on day 4, animals were gavaged with JD5037 3 mg/kg, MRI-1891 1 mg/kg, 3 mg/kg or vehicle, and the alcohol drinking session was repeated. Serum samples were collected from mandibular vein immediately after the last drinking session.

### Blood chemistry

Ethanol concentration was evaluated in serum samples with EnzyChromTM ethanol assay kit (BioAssay Systems) and plasma ghrelin was determined using commercial sandwich ELISA assays (Cayman Chemical). Samples were measured with the Spectra MAX 190 microplate reader (Molecular Devices, USA).

### Quantitative real-time PCR

Samples were prepared as described before [20]. Quantitative real-time PCR was performed by a QuantStudio 3 real time PCR system in a sample containing TaqMan™ Fast Advanced Master Mix, 25 ng of single stranded cDNA transcript and QuantiTect primers against mouse cannabinoid receptor 1 (Mm01212171_s1) and mouse S18 (Mm03928990_g1) as a housekeeping gene (Thermo Fisher Scientific, USA).

### Endocannabinoid measurement

Endocannabinoids were extracted and quantified by liquid chromatography–tandem mass spectrometry (LC-MS/MS) as described earlier [20].

### Tamoxifen injection

Six-week-old mice were injected subcutaneously (s.c.) with tamoxifen in a dose of 2 mg per mouse for 5 consecutive days (10 mg total dose) or its solvent corn oil in a volume of 100 µL as described [30]. Four weeks later animals were subjected to the alcohol test.

### Nodose ganglion injections with saporin conjugates

The procedure was performed as previously described [31]. 250 ng/ml of CB1-saporin conjugate or blank saporin were loaded into a NanofilTM 36 G beveled needle (World Precision Instruments, USA) using 10 μL 33 G Neuros Syringe (Hamilton, USA). The NanoFilTM injection holder was guided by the micromanipulator P-10 (Miller Design, USA) into the proximity of the nodose ganglion until the needle pierced it. For each nodose ganglion, a total 0.5 µL volume was delivered at 0.2 µL/min using a NanofilTM 10 ml syringe (World Precision Instruments, USA) mounted on a microinjection syringe pump World Precision Instruments, model MICRO2T (USA).

### CB_1_R binding assay

Binding affinity of MRI-1891 enantiomers to mouse brain membranes was determined by radioligand displacement assays using [^3^H]CP55,940 (Perkin-Elmer; USA) as described [32]. *Ki* values were calculated from the Cheng-Prusoff equation.

### Drugs

JD5037 [33] and MRI-1891 [34] enantiomers were synthesized in-house. Chemicals were obtained from Millipore Sigma (USA) except for blank saporin and CB1-saporin (Advanced Targeting Systems, USA), ethanol (The Warner Graham Company, USA) and saline (Avantor, USA). Stock solutions of JD5037, MRI-1891 and PF-5190457 were made in DMSO (6 mg/mL). Drugs were further dissolved in DMSO:Tween80:saline (5:2:93) before the administration. Protease inhibitors were prepared as described before [20]. Tamoxifen was suspended in ethanol (100 mg in 0.5 mL) of ethanol, vortexed and sonicate for 5 min at 37˚C until dissolved. Corn oil (4.5 mL) was added to make tamoxifen stock solution (20 mg/mL). The volume of 100 µL of tamoxifen (2 mg) or its vehicle was injected subcutaneously into the animal.

### Quantification and statistical analyses

Values are presented as mean ± SEM, with the number of replicates and the level of significance reported in figures, figure legends and in the Supplementary Material. A biological replicate (*n*) was defined as a mouse or its tissue from in vivo studies. Mice were randomized based on their body weights, drinking and eating habits, which were measured for 3 days (drinking in the dark) or 4 days (two-bottle choice test) prior to the treatment. Animal procedures were not conducted in a blind fashion. However, to minimize the risk of bias and ensure the reproducibility of the study, animal procedures were performed in small cohorts by different experimenters. In addition, various aspects of the study (e.g., animal data collection, endocannabinoid measurements, RNAscope imaging) were assigned to different investigators. Analysis of blood samples for ghrelin and ethanol was performed in a blinded manner. The number of mice used in behavioral studies was based on our previous experience, which showed that 8-10 animals in each treatment group were sufficient to perform meaningful comparisons [17, 20]. An online sample size calculator, www.ClinCalc.com, was used to determine the minimum number of test animals needed to achieve adequate statistical power. The calculator indicated a sample of 22 animals was required to detect a significant difference between two groups with a binomial endpoint, anticipated incidence of 25% in group 1 and 75% in group 2, a false positive error rate (α) of 0.2, a false negative error rate (β) of 0.1, and a power of 80%. This calculator was used as a guideline only and was not used for specific sample size calculations for individual mouse strains. Sample size was dependent on the availability of animals through in-house breeding. For the safety of investigators and animal caretakers, the number of mice injected with saporin conjugates was limited to 6 by the ACUC in the animal study protocol. A total of 10 out of 822 mice undergoing the two-bottle choice test were excluded because the initial genotype data was found to be incorrect when re-examined after the experiment was completed (3 mice) or because of weight loss caused by malocclusion (5 mice) or persistent seizures (2 mice), as confirmed by a veterinarian.

Statistical data analysis was performed using GraphPad Prism version 10.1.1 for Windows (GraphPad Prism Software Inc.). Data were assumed to have normal distribution based on the D’Agostino & Pearson normality test. For smaller datasets (n < 6), the Shapiro-Wilk test of normality was applied. The two-tailed Student’s *t*-test was used to compare values between two groups for paired or unpaired data. An ordinary one-way ANOVA with Dunnett’s post-hoc test was applied for multiple groups. Time-dependent variables were analyzed using two-way ANOVA with Šídák’s or Tukey’s corrections when comparing one or multiple groups with the control. Data groups were automatically tested for the homogeneity of variance with either the F-test (for Student’s *t*-test), the Brown-Forsythe test (for one-way ANOVA) or Levene’s test (for two-way ANOVA). Where appropriate, Welch’s t-test or Brown-Forsythe ANOVA was used to correct for unequal variances. For some datasets that were not normally distributed, the two-sided Mann Whitney and Kruskal–Wallis tests were used to evaluate differences between two groups and multiple groups. Statistical tests were clearly indicated in the legend of each figure and in the Supplementary Material when significant differences between groups were reported. Differences were considered significant when *P* < 0.05. QuPath [35] version 0.5.1 and Fiji [36] were used for image analyses. The raw data points and corresponding estimates of variation (standard deviation and variance) for each data group are presented in the source data file.

## Results

### Deletion of CB_1_R from ghrelin-producing cells does not prevent JD5037-induced inhibition of VEI

**Fig. 1:**
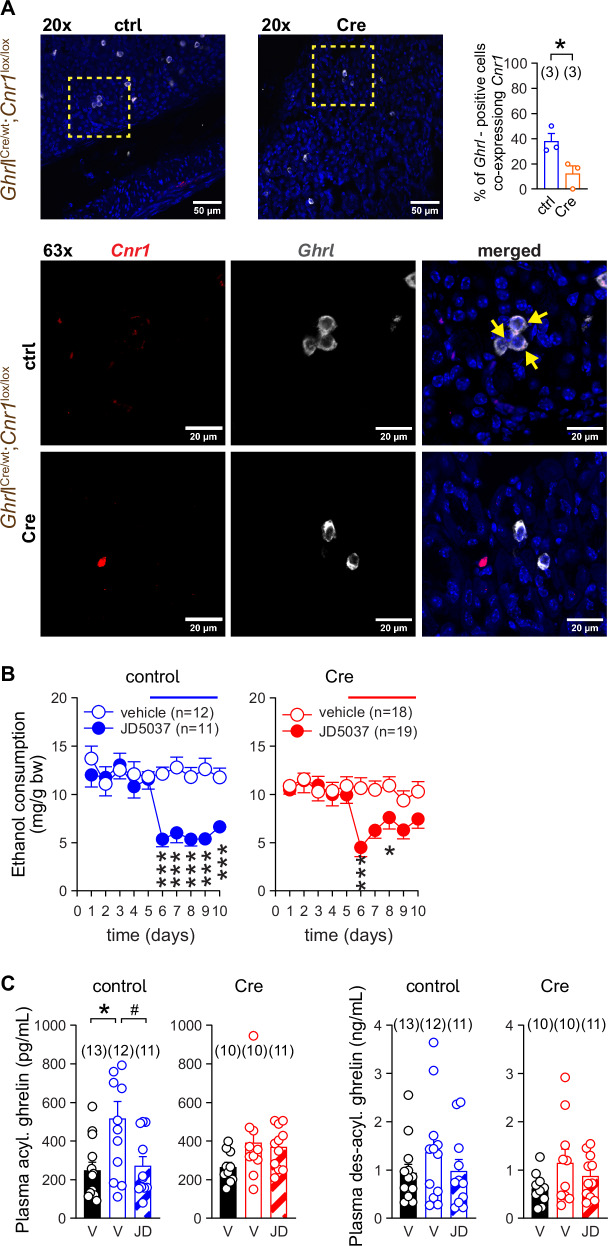
Deletion of CB_1_R from ghrelin-producing cells is necessary but not sufficient to occlude JD5037-induced inhibition of VEI. **A** RNAscope images of *Cnr1* and *Ghrl* expressions in the stomach sections from control (ctrl) (*Ghrl*^wt^;*Cnr1*^lox/lox^) and ghrelin-producing cell-specific *Cnr1*-deficient (Cre) (*Ghrl*^Cre^;*Cnr1*^lox/lox^) littermate mice. Yellow squares indicate areas from 20x images displayed at 63x magnification. Arrows indicate presence of *Cnr1* mRNA (red) in ghrelin producing cells (white). DAPI (blue) visualizes nuclear DNA. **B** The effect of CB_1_R blockade with JD5037 3 mg/kg p.o. on VEI in control (*Ghrl*^wt^;*Cnr1*^lox/lox^) and ghrelin-producing cell-specific *Cnr1*-deficient (Cre) (*Ghrl*^Cre^;*Cnr1*^lox/lox^) littermate mice. **C** Plasma acylated (acyl.) and unacylated (des-acyl.) ghrelin levels in control (*Ghrl*^wt^;*Cnr1*^lox/lox^) and ghrelin-producing cell-specific *Cnr1*-deficient (Cre) (*Ghrl*^Cre^;*Cnr1*^lox/lox^) littermate mice at the end of the 5-day treatment period. Values are obtained from ethanol-naïve animals (filled bars) and from ethanol-drinking mice (hatched and open bars), treated with vehicle (V) or JD5037 at a dose of 3 mg/kg p.o. (JD). Points and bars represent means ± SEM from indicated number of experiments. *P < 0.05, ***P < 0.001 by Student’s t-test (A) or by two-way ANOVA (B). The percentage of *Ghrl*-positive cells co-expressing *Cnr1* (A) was calculated as the average of three areas randomly selected from images at 20x magnification. The co-expression was verified at 63x magnification. Significant differences between corresponding values in ‘no ethanol’ group (filled column) and ethanol-drinking mice treated with the vehicle (open columns) (*P < 0.05) or between vehicle- and JD5037-treated alcohol groups (open and hatched columns, respectively) (^#^P < 0.05) by Brown-Forsythe ANOVA (bar graph 1 in C). Ordinary one-way ANOVA was used for other bar graphs (C). Raw data points and the associated estimates of variation are accessible in the source data file.

We first generated transgenic mice lacking CB_1_R in ghrelin-producing cells by crossing mice with *loxP* sites flanking the CB_1_R gene [37] (*Cnr1*^*lox/lox*^) with mice expressing Cre recombinase in ghrelin-producing cells via the ghrelin peptide (*Ghrl*) promoter/enhancer sequences [23]. Successful deletion of the CB_1_R gene and generation of the desired transgenic phenotypes, i.e., tissue-specific CB_1_R knock-out (KO) (*Ghrl*^Cre^;*Cnr1*^*lox/lox*^) and littermate control (*Ghrl*^wt^;*Cnr1*^*lox/lox*^), was confirmed by PCR (Figure S1A) and RNA in situ hybridization. Using the latter we found low mRNA expression of *Cnr1* in a fraction of ghrelin-producing gastric mucosal cells in control mice, consistent with earlier findings [20, 23], and its absence in KO mice (Fig. 1A).

Male control mice with access to water and 15% ethanol solutions showed somewhat higher intake and preference for ethanol than their conditional KO littermates, resulting in an average daily intake of 11.8 ± 0.7 mg ethanol/g body weight (n = 23) and 10.3 ± 0.6 mg ethanol/g body weight (n = 37), respectively (Table S1). VEI remained unaffected by vehicle treatment but was sharply reduced in control mice with daily oral doses of 3 mg/kg of the peripherally-restricted CB_1_R inverse agonist JD5037, while the KO mice exhibited a weaker response to the drug (Fig. 1B).

Ethanol-drinking control mice exhibited elevated plasma levels of octanoyl-ghrelin and tended to increase its inactive precursor desacyl-ghrelin. Consistent with our earlier observations, JD5037 selectively blunted the increase in octanoyl ghrelin in control mice without affecting desacyl ghrelin levels (Fig. 1C). Unlike control mice, KO mice did not show significant changes in plasma octanoyl-ghrelin levels due to ethanol drinking or JD5037 treatment (Fig. 1C). The lack of increase in plasma octanoyl-ghrelin and the failure of JD5037 to reduce it in ethanol-drinking KO mice may signify an EC tone deficiency in ghrelin-producing cells due to the absence of CB_1_R expression. Despite the unchanged plasma ghrelin level, JD5037 was still able to moderately inhibit ethanol drinking in KO mice (Fig. 1B), which points to the involvement of additional peripheral CB_1_R in cell types other than ghrelin-producing cells to drive VEI.

### CB_1_R on a subset of advillin^+^ sensory neurons mediates JD5037-induced inhibition of VEI

**Fig. 2:**
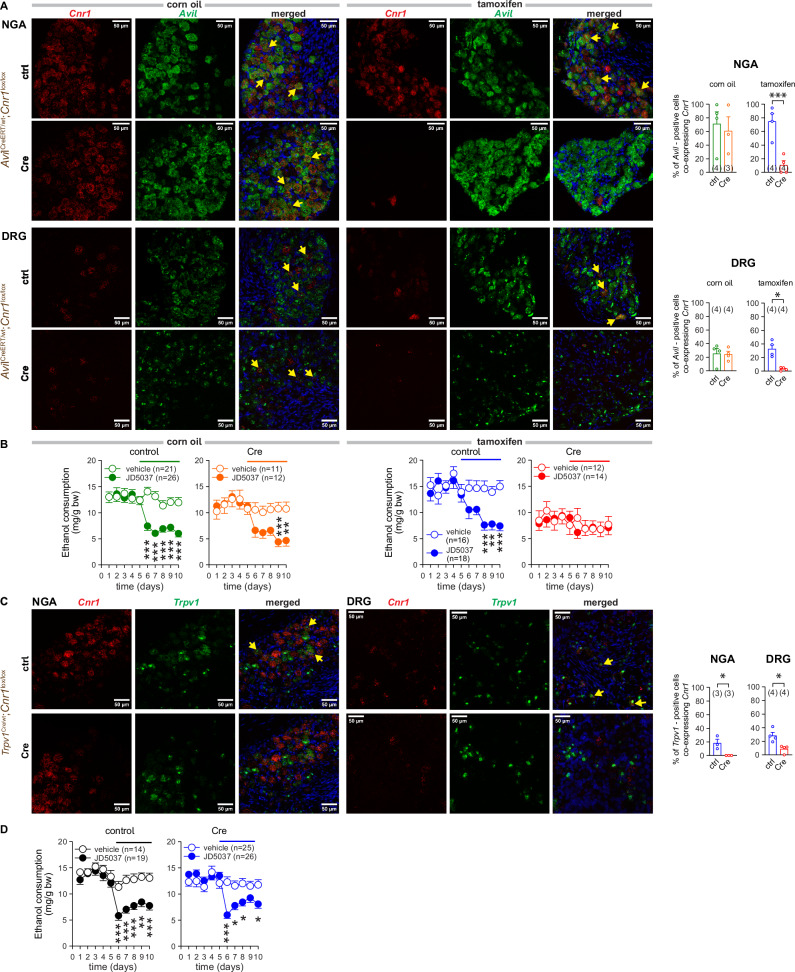
Deletion of CB_1_R from advillin^+^ sensory afferent neurons modulates VEI. **A** RNAscope images of *Cnr1* and *Avil* expressions in NGA and DRG tissue sections from Cre-negative (control, ctrl) (*Avil*^wt^;*Cnr1*^lox/lox^) and Cre-positive (Cre) (*Avil*^CreERT^;*Cnr1*^lox/lox^) littermate mice receiving corn oil or tamoxifen. Co-localization was assessed by overlaying 2 channels. Arrows indicate examples of co-localization of mRNA for CB_1_R mRNA (red) and advillin (green). DAPI (blue) visualizes nuclear DNA. **B** The effect of CB_1_R blockade with JD5037 3 mg/kg p.o. on VEI in Cre-negative (control) (*Avil*^wt^;*Cnr1*^lox/lox^) and Cre-positive (Cre) (*Avil*^CreERT^;*Cnr1*^lox/lox^) littermate mice receiving corn oil or tamoxifen. **C** *Cnr1* and *Trpv1* expression as visualized by fluorescent RNAscope imaging of NGA and DRG tissue sections from Cre-negative (control, ctrl) (*Trpv1*^wt^;*Cnr1*^lox/lox^) and Cre-positive (Cre) (*Trpv1*^Cre^;*Cnr1*^lox/lox^) littermate mice. Co-localization was assessed by overlaying 2 channels. Arrows show examples of co-localization of mRNA for CB_1_R (red) and Trpv1 (green) and DAPI (blue). **D** The effect of CB_1_R blockade with JD5037 3 mg/kg p.o. on VEI in control (*Trpv1*^wt^;*Cnr1*^lox/lox^) and Trpv1 cell-specific *Cnr1*-deficient (Cre) (*Trpv1*^Cre^;*Cnr1*^lox/lox^) littermate mice. Points and bars represent means ± SEM from the indicated number of experiments. *P < 0.05, **P < 0.01, ***P < 0.001 by Student’s t-test for unpaired samples (upper bar graph in A), by Welch’s t-test (bottom bar graph in A) or by two-way ANOVA (B, D), or by Mann Whitney U test (C). Raw data points and the associated estimates of variation are accessible in the source data file.

Peripheral sensory neurons detect and transmit various interoceptive and exteroceptive signals to the brain via dorsal root ganglia (DRG) and nodose ganglia (NGA). Both NGA and DRG neurons uniquely express the actin-binding protein advillin, encoded by the *Avil* gene, during development and into adulthood [38]. Both NGA and DRG neurons also express TRPV1 [39], the receptor for capsaicin, a compound used to achieve afferent vagal denervation [20]. To identify sites of peripheral CB_1_R signaling beyond ghrelin-producing cells that promote VEI, *i.e*., sensory neuronal *vs* extra-neuronal sites, we used *Avil*^CreERT2/wt^ mice to create a conditional knockout mouse line, in which the fate of CB_1_R in *Avil*-expressing sensory neurons is controlled by a tamoxifen-inducible Cre recombinase driven by the Avil gene promoter. To achieve this targeted deletion, six-week-old transgenic mice (*Avil*^CreERT2^;*Cnr1*^*lox/lox*^) and their control littermates (*Avil*^wt^;*Cnr1*^*lox/lox*^) mice were injected with tamoxifen daily and were then subjected to the two-bottle choice test. To control the possible effect of dormant Cre on *Cnr1* expression, other cohorts of mice received the solvent for tamoxifen, corn oil. The successful generation of transgenic phenotypes was confirmed by genotyping (Figure S1B) and with RNAscope®. There was an abundant co-expression of *Cnr1* and *Avil* mRNA in NGA and less abundant in DRG in all transgenic mice treated with corn oil and in *Avil*^wt^;*Cnr1*^*lox/lox*^ control mice injected with tamoxifen. In contrast, *Cnr1* mRNA was not detectable in *Avil*-positive cell bodies in either of the tissues taken from *Avil*^CreERT2^;*Cnr1*^*lox/lox*^ mice receiving tamoxifen (Fig. 2A).

Selective tamoxifen-inducible deletion of CB_1_R from *Avil*-expressing neurons of *Avil*^CreERT2^;*Cnr1*^*lox/lox*^ mice resulted in a marked reduction in baseline ethanol intake (Table S1) and blunted the effect of JD5037 treatment in ethanol-drinking mice (Fig. 2B), suggesting a unique role of this subset of sensory afferent neurons in controlling such intake. The presence of dormant *Cre* in *Avil*-positive neurons of corn oil-injected mice did not affect ethanol drinking (Fig. 2B, Table S1). Chemical denervation by capsaicin eliminated the inhibition of VEI by antagonists of CB_1_R and Ghsr [20]. By crossing *Cnr1*^*lox/lox*^ with the *Trpv1*^Cre/wt^ transgenic mice (Figure S1C) we successfully eliminated CB_1_R from a small population of Trpv1-coexpressing NGA and DRG neurons (Fig. 2C). Unexpectedly, this deletion did not affect JD5037’s inhibitory effect on VEI (Fig. 2D, Table S1), suggesting that CB_1_R on Trpv1^+^ sensory afferents are not involved in the control of VEI in mice.

### Deletion of CB_1_R from NGA but not DRG neurons abolishes JD5037-induced inhibition of VEI

**Fig. 3:**
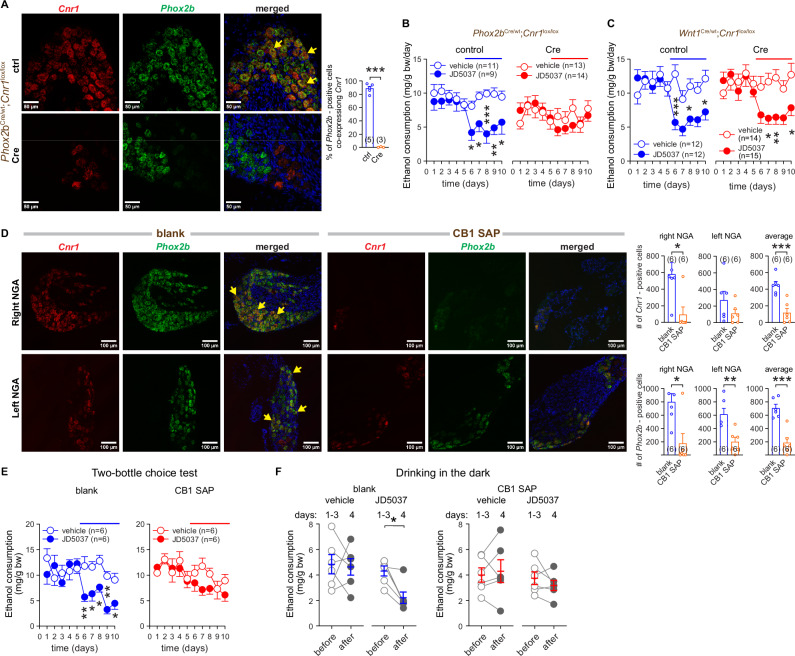
Deletion of CB_1_R from NGA neurons but not DRG neurons is sufficient to abolish JD5037-induced inhibition of VEI. **A** *Cnr1* and *Phox2b* expression as visualized by fluorescent RNAscope imaging of NGA tissue sections from control (ctrl) (*Phox2b*^wt^;*Cnr1*^lox/lox^) and Cre-positive (Cre) (*Phox2b*^Cre^;*Cnr1*^lox/lox^) littermate mice. Co-localization was assessed by overlaying 2 channels. Arrows show examples of co-localization of mRNA for CB_1_R (red) and Phox2b (green). DAPI (blue) visualizes nuclear DNA. **B** The effect of CB_1_R blockade with JD5037 3 mg/kg p.o. on VEI in control (*Phox2b*^wt^; *Cnr1*^lox/lox^) and tissue-specific *Cnr1*-deficient (Cre) (*Phx2b*^Cre^; *Cnr1*^lox/lox^) littermate mice. **C** The effect of CB_1_R blockade on VEI in control (*Wnt1*^wt^; *Cnr1*^lox/lox^) and tissue-specific *Cnr1*-deficient (Cre) (*Wnt1*^Cre^;*Cnr1*^lox/lox^) littermate mice. **D** *Cnr1* and *Phox2b* expression in blank saporin (Blank) and CB1-saporin (CB1 SAP)-injected mouse NGA tissue sections as visualized by fluorescent RNAscope imaging. Co-localization was assessed by overlaying 2 channels. Arrows show examples of co-localization of mRNA for CB_1_R (red) and Phox2b (green). DAPI (blue) visualizes nuclear DNA. For additional images, see Figure S2. **E** The effect of JD5037 3 mg/kg p.o. on VEI in mice undergoing the two-bottle paradigm, whose NGA were bilaterally injected with blank saporin (blank) and CB1-saporin (CB1 SAP). **F** The effect of JD5037 3 mg/kg p.o. on VEI in mice tested in the drinking in the dark paradigm, whose NGA were bilaterally injected with blank saporin (blank) and CB1-saporin (CB1 SAP). Points and bars represent means ± SEM from the indicated number of experiments (A-E). Drinking behavior in individual animals is expressed as points before (average of days 1–3) and after treatment on day 4 (F). *P < 0.05, **P < 0.01, ***P < 0.001 by the Welch’s t-test (A), Student’s t-test for unpaired (D) and paired samples (F), one-way ANOVA (bar graphs in E), or by two-way ANOVA (B, C, E). Raw data points and the associated estimates of variation are accessible in the source data file.

CB_1_R is expressed in NGA [10, 11, 24] and DRG [26] and the corresponding vagal and DRG sensory terminals project to the GI tract [7]. To remove CB_1_R selectively from the NGA, we crossed *Cnr1*^*lox/lox*^ mice with *Phox2b*^Cre/wt^ mice, which targets epibronchial placodes-derived cells, including vagal afferents [40], but not DRG neurons. As shown earlier, *Wnt1* mRNA is expressed in DRG neurons, but not detectable in nodose ganglia [7]. To remove CB_1_R selectively from DRG neurons, we crossed *Cnr1*^*lox/lox*^ mice with *Wnt1*^Cre/wt^ mice [41], which targets cells derived from the neuronal crest, including spinal afferents. The resulting mutant mice, *i*.*e*., NGA-specific CB_1_RKO mice (*Phox2b*^Cre^;*Cnr1*^*lox/lox*^) and their control littermates (*Phox2b*^wt^; *Cnr1*^*lox/lox*^) and DRG-specific CB_1_RKO mice (*Wnt1*^Cre^;*Cnr1*^*lox/lox*^) and their controls (*Wnt1*^wt^;*Cnr1*^*lox/lox*^) were validated by genotyping (Figures S1D and S1E). Also, there was an abundant co-expression of *Cnr1* and *Phox2b* in nodose ganglia of control (*Phox2b*^wt^*Cnr1*^*lox/lox*^) mice and disappearance of *Cnr1* from the same tissue of KO (*Phox2b*^Cre^;*Cnr1*^*lox/lox*^) mice (Fig. 3A).

The average daily intake of ethanol was comparable between *Wnt1*^wt^;*Cnr1*^*lox/lox*^ and *Wnt1*^Cre^;*Cnr1*^*lox/lox*^ mice (Table S1) and tended to be lower in *Phox2b*^Cre^;*Cnr1*^*lox/lox*^ than in *Phox2b*^wt^;*Cnr1*^*lox/lox*^ mice (Table S1). In the two control mouse strains JD5037 was similarly effective in inhibiting VEI (Fig. 3B and C, left panels). The deletion of CB_1_R from vagal afferents expressing Phox2b was sufficient to abolish the inhibitory effect of JD5037 (Fig. 3B, right panel). In contrast, the drug remained effective after knocking out CB_1_R from DRG (Fig. 3C, right panel).

### Selective ablation of CB_1_R^+^ NGA neurons blunt JD5037-induced inhibition of VEI

As aberrant expression of the neuronal markers we used above may complicate the interpretation of the results [42], we selectively ablated CB_1_R-expressing vagal afferent fibers of the GI tract by injecting the ribosomal inhibitor saporin [43] conjugated to CB_1_R antibody (CB1 SAP) or unconjugated saporin (Blank) into the left and right NGA of C57BL/6 J mice. The effectiveness of this approach is evidenced by partial (in ganglia of 2 out of 6 mice) to nearly complete disappearance (in ganglia from the remaining 4 mice) of both *Cnr1* and *Phox2b* mRNA from the NGA of CB1 SAP-injected mice compared to the control mice receiving Blank (Fig. 3D, Figure S2). *Cnr1* expression appeared to be higher in the right NGA, which resulted in a more effective ablation of CB_1_R-expressing vagal afferent fibers by CB1 SAP (Fig. 3D). Bilateral vagal applications of Blank and CB1 SAP did not affect baseline VEI (Table S1). However, CB1 SAP treatment blunted the inhibitory effect of JD5037 to non-significant levels in both the ‘two bottles/free choice’ and the ‘drinking in the dark’ ethanol drinking paradigms, which parallels the degree of vagal deafferentation of the NG CB_1_R^+^ neurons (Fig. 3E and F). This confirms the role of CB_1_R^+^ NGA vagal afferents in regulating VEI by mice.

### Validation of CB_1_R as the target of JD5037 for suppressing VEI

**Fig. 4:**
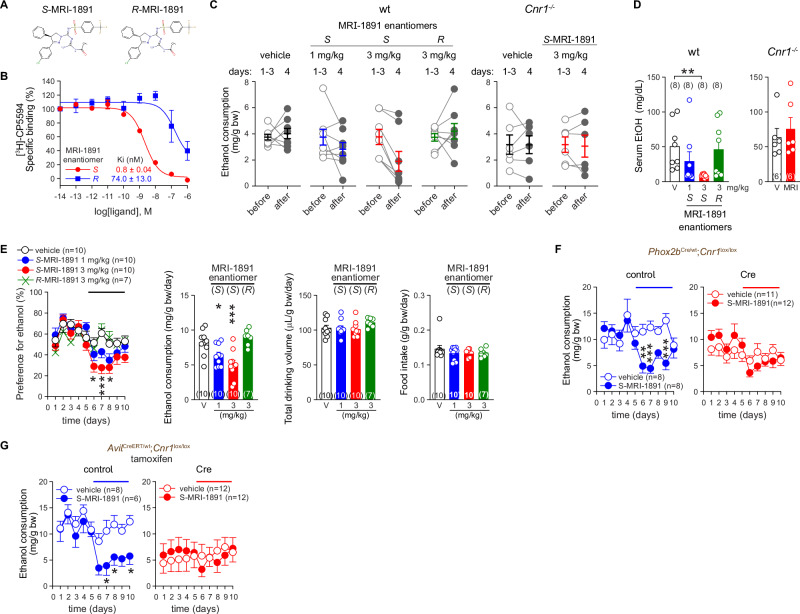
Biased non-brain penetrant CB_1_R antagonist MRI-1891 regulates VEI through peripheral sensory neuronal CB_1_R. **A** Chemical structures of MRI-1891 enantiomers. **B** CB_1_R binding affinity of MRI-1891 enantiomers as determined from displacement isotherms of [^3^H]-CP55940 in crude mouse brain membranes. **C** The effect of oral administration of *S* and *R* enantiomers of MRI-1891 on VEI in wild-type (wt) and CB1R-deficient (*Cnr1*^*-/-*^) littermate mice in the drinking-in-the-dark paradigm. **D** The corresponding serum ethanol in wild-type (wt) and CB1R-deficient (*Cnr1*^*-/-*^) littermates subjected to the drinking-in-the dark paradigm and treated with *S* and *R* enantiomers of MRI-1891 (MRI) or vehicle (V). **E** The effect of CB_1_R blockade with *S* and *R* enantiomers of MRI-1891 on VEI in C57BL/6 J mice in the two-bottle choice test. **F** The effect of CB_1_R blockade with *S*-MRI-1891 3 mg/kg p.o. on VEI in control (*Phox2b*^wt^; *Cnr1*^lox/lox^) and tissue-specific *Cnr1*-deficient (Cre) (*Phox2b*^Cre^;*Cnr1* ^lox/lox^) littermate mice. **G** The effect of CB_1_R blockade with *S*-MRI-1891 3 mg/kg p.o. on VEI in control (*Avil*^wt^;*Cnr1*^lox/lox^) and tissue-specific *Cnr1*-deficient (Cre) (*Avil*^CreERT^;*Cnr1*^lox/lox^) littermate mice pretreated with tamoxifen. Points and bars represent means ± SEM from the indicated number of experiments. Chiral selectivity of MRI-1891 enantiomers to CB_1_R (B) was performed three times in triplicates. Drinking behavior in individual animals is expressed as points before (average of days 1–3) and after treatment on day 4 (C). Bars and points in (E) show daily and 5-day drinking average, respectively *P < 0.05, **P < 0.01, ***P < 0.001 by Student’s t-test for paired (C) or unpaired samples (D), by Kruskal–Wallis test (D), by one-way ANOVA (bar graphs in E) or by two-way ANOVA (E, F, G). Raw data points and the associated estimates of variation are accessible in the source data file.

JD5037 is a highly potent CB_1_R antagonist/inverse agonist with no known off-target effects at the doses used here. Nevertheless, to further validate CB_1_R as its target for inhibiting VEI, we tested another peripheral CB_1_R antagonist/inverse agonist in our VEI paradigms. *S*-MRI-1891, invented in our laboratory [32], is a peripherally restricted CB_1_R antagonist/inverse agonist with sub-nanomolar potency for binding to CB_1_R, whereas it’s enantiomer *R*-MRI-1891 has 3 orders of magnitude lower CB_1_R binding potency (Fig. 4A and B) [32].

MRI-1891 markedly inhibited alcohol intake in mice and its effect was isomer-specific in two experimental models. In the drinking-in-the-dark paradigm, oral administration of the active (*S*) isomer of MRI-1891 inhibited alcohol consumption in wild-type mice in a dose-dependent manner, whereas its inactive (*R*) isomer had no such effect. Furthermore, this effect was CB_1_R-dependent as it did not occur in *Cnr1*^*-/-*^ [44] mice. (Fig. 4C). This pattern was corroborated by corresponding changes in serum alcohol levels (Fig. 4D), indicating that the drug does not influence the rate of alcohol metabolism. The same trend was evident when using the two-bottle choice test (Fig. 4E). High alcohol preference and intake in male C57BL/6 J mice (Table S1) remained unchanged with daily gavage of either vehicle and *R*-MRI-1891, while *S*-MRI-1891 significantly reduced alcohol preference and intake without affecting total liquid or food consumption (Fig. 4E). The deletion of CB_1_R from *Phox2b-*expressing neurons or *Avil*-expressing neurons in *Phox2b*^Cre^;*Cnr1*^*lox/lox*^ and *Avil*^CreERT2^;*Cnr1*^*lox/lox*^ mice abolished the effect of *S*-MRI-1891 (Fig. 4F and G, respectively). This finding indicates that CB_1_R expressed by Phox2b^+^ and advillin^+^ subsets of peripheral sensory afferent neurons is the common target of different CB_1_R antagonists for suppressing VEI.

### Peripheral CB_1_R antagonists require CB_1_R in NGA neurons to inhibit VEI in female mice

Although CB_1_R is known to regulate alcohol consumption in female mice [28], the efficacy and specific peripheral targets of CB_1_R antagonists, compared to males, remain unclear. In the two-bottle choice test, wild-type females consumed more ethanol than *Cnr1*^*-/-*^ mice (Table S1). Oral administration of JD5037 (3 mg/kg daily) significantly reduced VEI in wild-type mice, with no effect in *Cnr1*^*-/-*^ counterparts (Figure S3A). Similar results were observed with MRI-1891 in the drinking-in-the-dark paradigm. In *Phox2b*^wt^;*Cnr1*^*lox/lox*^ mice, MRI-1891 reduced ethanol intake in an isomer-specific manner, whereas the effect of *S*-MRI-1891 was abolished in *Phox2b*^Cre^;*Cnr1*^*lox/lox*^ mice (Figure S3B). These behavioral changes were consistent with corresponding serum alcohol levels (Figure S3C). This confirms the role of CB_1_R^+^ NGA vagal afferents in regulating VEI in both sexes.

### CB_1_R on Gpr65^+^ subset of NGA neurons is the target of JD5037 for suppressing VEI

**Fig. 5:**
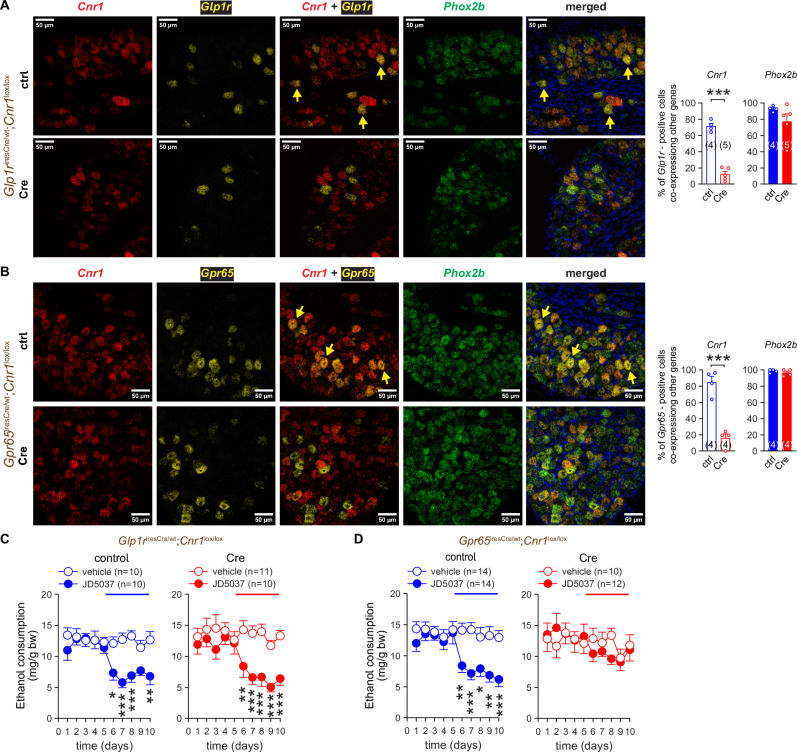
Deletion of CB_1_R from Gpr65^+^ but not Glp1R^+^ NGA neurons blunts JD5037-induced inhibition of VEI. **A**, **B** *Cnr1, Phox2b* and *Glp1r* (A) *or Cnr1, Phox2b* and*’Gpr65* (B) as visualized by fluorescent RNAscope imaging of NGA tissue sections from Glp1r^+^ cell-specific *Cnr1*-deficient (Cre) (*Glp1r*^iresCre^;*Cnr1*^lox/lox^) (A) and Gpr65^+^ cell-specific *Cnr1*-deficient (Cre) (*Gpr65*^iresCre^;*Cnr1*^lox/lox^) (B) mice and their respective control (ctrl) littermates (*Glp1r*^wt^;*Cnr1*^lox/lox^ *Gpr65*^wt^;*Cnr1*^lox/lox^). Co-localization was assessed by overlaying 3 channels. Arrows show mRNA for CB_1_R (red), Phox2b (green) and either Glp1r (A) or Gpr65 (B) (yellow). DAPI (blue) visualizes nuclear DNA. **C**, **D** The effect of CB_1_R blockade with JD5037 3 mg/kg p.o. on VEI in *Glp1r*^iresCre^;*Cnr1*^lox/lox^ mice (Cre) (C) and *Gpr65*^iresCre^;*Cnr1*^lox/lox^ (Cre) (D) mice and their respective control littermate mice *Glp1r*^wt^;*Cnr1*^lox/lox^ and *Gpr65*^wt^;*Cnr1*^lox/lox^. Points and bars represent means ± SEM from the indicated number of experiments. *P < 0.05, **P < 0.01, ***P < 0.001 by Student’s t-test for unpaired samples (A, B) or by two-way ANOVA (C, D). Raw data points and the associated estimates of variation are accessible in the source data file.

Afferent NGA projections of Phox2b^+^ neurons have been detected in the muscular and mucosal layers of the GI tract. These projections represent two non-overlapping populations of vagal afferents whose genetic markers are *Glp1r* and *Gpr65*, respectively. To determine the contribution of these two populations of sensory neurons to CB_1_R-mediated VEI, we sought to eliminate CB_1_R expression from the mucosal or the muscular layer by crossing *Cnr1*^*lox/lox*^ mice with the *Glp1r*^iresCre^ [22] and *Gpr65*^iresCre^ [21] mouse lines, respectively. The resulting mutant mice, *i*.*e*., muscular layer-specific CB_1_RKO (*Glp1r*^iresCre^;*Cnr1*^*lox/lox*^) and mucosal layer-specific CB_1_RKO mice (*Gpr65*^iresCre^;*Cnr1*^*lox/lox*^) and respective control littermates (*Glp1*r^wt^;*Cnr1*^*lox/lox*^and *Gpr65*^wt^;*Cnr1*^*lox/lox*^) were assessed by genotyping (Figures S1F and S1G) and confirmed using RNAscope®. We found abundant mRNA co-expression of *Cnr1* with either *Phox2b* or *Glp1r* in NGA cell bodies of control (*Glp1r*^wt^;*Cnr1*^*lox/lox*^) mice and an absence of *Cnr1* in the same tissue of KO (*Glp1r*^iresCre^;*Cnr1*^*lox/lox*^) mice (Fig. 5A). Likewise, *Cnr1* was co-expressed with *Phox2b* and *Gpr65* mRNA in NGA of *Gpr65*^wt^;*Cnr1*^*lox/lox*^ mice but was absent in NGA obtained from G*lp1r*^iresCre^;*Cnr1*^*lox/lox*^ mice (Fig. 5B). The average daily intake of ethanol was similar among the transgenic strains (Table S1). Knocking out CB_1_R in *Glp1r*-expressing vagal afferents of the muscular layer did not affect the inhibitory effect of JD5037 (Fig. 5C). In contrast, the deletion of CB_1_R in vagal afferents expressing *Gpr65* was sufficient to abolish JD5037-mediated inhibition of VEI (Fig. 5D).

Because deletion of CB_1_R from *Avil*-expressing sensory afferent neurons had a strong effect on ethanol drinking in mice, we tested whether advillin^+^ and Gpr65^+^ neurons form distinct subsets of NGA neurons with unique roles in controlling alcohol intake. As visualized by fluorescent RNAscope imaging of NGA tissue sections from C57BL6/J mice, advillin^+^ neurons constitute a significant pool of neurons (Figure S4). Approximately 20.62 ± 5.40% of all NGA cell bodies express *Avil* and only 1.39 ± 0.54% of cells express *Gpr65* (n = 5). *Gpr65* appears exclusively on *Avil*-expressing neurons (92.42 ± 3.58%; n = 5), indicating that *Avil*-expressing neurons reach the mucosal layer of the GI tract along with Gpr65^+^ neurons. The remaining majority of advillin^+^ cell bodies (93.45 ± 1.71%; n = 5) showed no detectable levels of *Gpr65* (Figure S4).

### Ghsr deletion from Phox2b^+^ NGA neurons blunts the inhibition of VEI by both Ghsr and CB_1_R antagonists

**Fig. 6:**
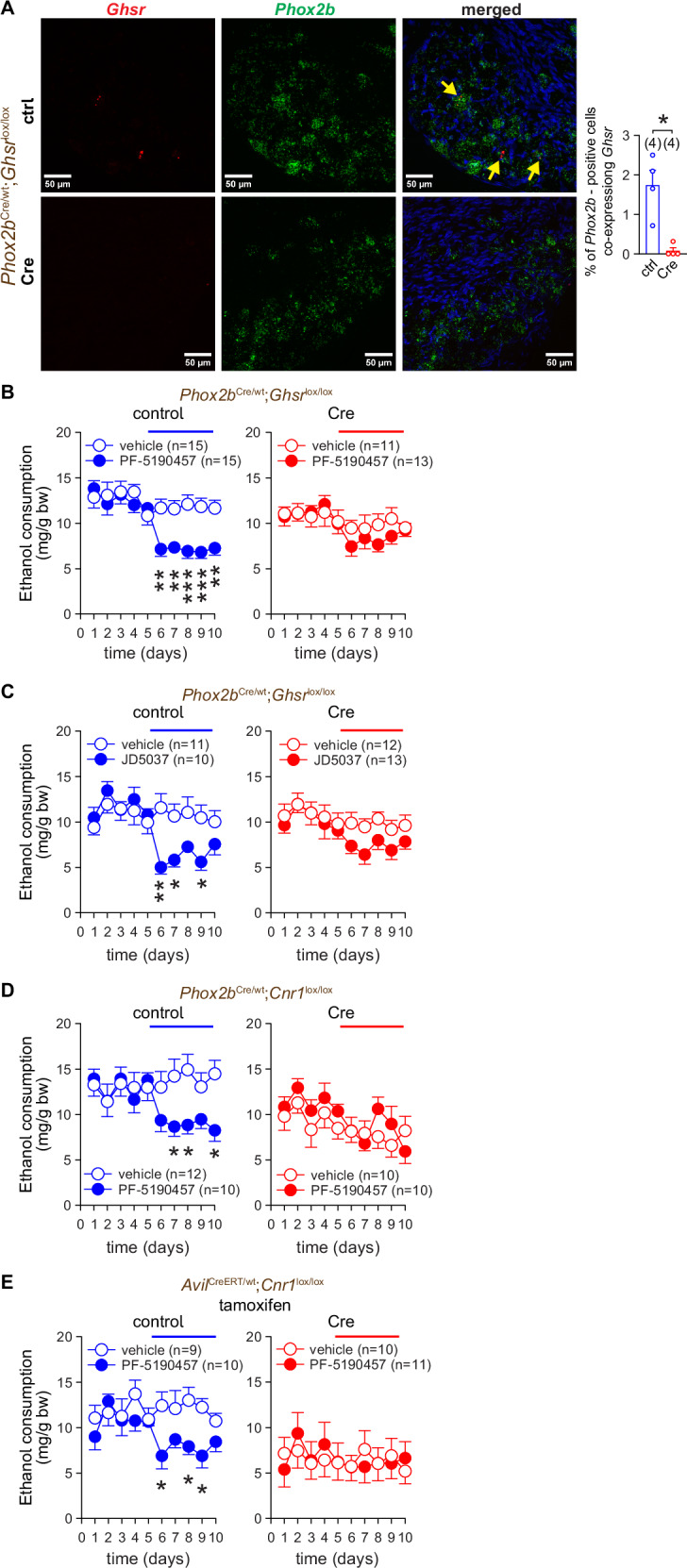
CB_1_R and Ghsr expressed on Phox2b^+^ NGA neurons mediate the inhibitory effects of their respective antagonists on VEI. **A** *Ghsr* and *Phox2b* expression as visualized by fluorescent RNAscope imaging of NGA tissue sections from control (ctrl) (*Phox2b*^wt^;*Ghsr*^lox/lox^) and Cre-positive (Cre) (*Phox2b*^Cre^;*Ghsr*^lox/lox^) littermate mice. Co-localization was assessed by overlaying 2 channels. Arrows show examples of co-localization of mRNA for *Ghsr* (red) and *Phox2b* (green). DAPI (blue) visualizes nuclear DNA. **B**, **C** The effect of peripheral Ghsr antagonist PF-5190457 3 mg/kg p.o. (B) and peripheral CB_1_R antagonist JD5037 3 mg/kg p.o. (C) on VEI in control (*Phox2b*^wt^;*Ghsr*^lox/lox^) and NGA-specific *Ghsr*-deficient (Cre) (*Phox2b*^Cre^;*Ghsr*^lox/lox^) littermate mice. **D**, **E** The effect of the peripheral Ghsr antagonist PF-5190457 3 mg/kg p.o. on alcohol intake in Cre-positive (Cre) NGA-specific CB_1_RKO mice (*Phox2b*^*Cre*/wt^;*Cnr1*^lox/lox^) (D) and Advillin^+^ cell-specific CB_1_R-deficient mice (*Avil*^CreERT^;*Cnr1*^lox/lox^) (E) and in their respective control littermate mice (Phox*2b*^wt/wt^;*Cnr1*^lox/lox^ and *Avil*^wt^;*Cnr1*^lox/lox^). Points and bars represent means ± SEM from the indicated number of experiments. *P < 0.05, **P < 0.01, ***P < 0.001 by the Mann Whitney U test (A) or by two-way ANOVA (B-E). Raw data points and the associated estimates of variation are accessible in the source data file.

Ghsr is localized in many brain regions associated with pleasure, food and alcohol reward [45, 46], as well as outside the brain [20, 47], we sought to test whether Ghsr deletion from NGA neurons is sufficient to lower VEI and limit the effect of the CB_1_R antagonist on this readout. For this purpose, we created *Ghsr*^*lox/lox*^ mice, which were then bred with *Phox2b*^Cre/wt^ mice to obtain NGA-GhsrKO (*Phox2b*^Cre^;*Ghsr*^*lox/lox*^) mice and control littermates (*Phox2b*^wt^;*Ghsr*^*lox/lox*^), verified by genotyping (Figure S1H). RNAscope® images of NGA sections from control mice revealed a very discrete population of Phox2b^+^ neurons co-expressing *Ghsr* mRNA, which was absent from NGA-GhsrKO mice (Fig. 6A). When subjected to a two-bottle choice test, NGA-GhsrKO mice tended to consume less ethanol than their control littermates (Table S1). Ethanol consumption was inhibited by the ghrelin receptor antagonist PF-5190457 in the control mice, whereas NGA-GhsrKO mice no longer responded to PF-5190457 treatment (Fig. 6B). NGA-GhsrKO mice also became less sensitive to JD5037 (Fig. 6C). Reciprocally, mice deficient in CB_1_R in Phox2b^+^ (Fig. 6D), or in advillin^+^ sensory afferent neurons (Fig. 6E) no longer responded to PF-5190457.

### Alcohol drinking does not affect *Cnr1* expression in nodose ganglia or food intake

*Cnr1* and *Phox2b* expressions in the NGA did not differ between alcohol-naïve and alcohol-exposed groups, as measured by RNAscope (Figure S5A) and RT-qPCR (Figure S5B). Similarly, NGA endocannabinoid levels were unchanged (Figure S5C). In contrast, alcohol-drinking mice exhibited increased oleoylethanolamide (OEA) levels in the NGA in the two-bottle choice test, an effect not seen in other tissues (Figure S5D).

The average daily intake of standard chow in alcohol-naïve mice and those exposed to 15% ethanol during the two-bottle choice test was 137.88 ± 7.71 mg/g bw (n = 5) and 146.42 ± 9.67 mg/g bw (n = 5), respectively. Drug treatments in multiple transgenic mouse lines had no significant impact on food intake (Table S2).

## Discussion

The vagus nerve is known to transmit the caloric and rewarding values of food from the gut to the brain [6, 7, 9–11], and has been investigated as a potential target to treat AUD via incretins [18] and GI hormones [48]. This study provides new insights into the regulation of alcohol drinking behavior by peripheral ECs through the gut-brain axis, identifying CB_1_R on vagal sensory afferent neurons as critical regulators of VEI in male and female mice and implicates a subset of vagal afferent neurons expressing advillin and Gpr65 in the modulation of VEI. The study also reveals a crosstalk between vagal sensory CB_1_R and Ghsr, indicating that the drive to drink alcohol depends not only on CB_1_R on ghrelin-producing cells that control acyl-ghrelin tone [20], but also on CB_1_R directly expressed on vagal afferent terminals.

Our results indicate that CB_1_R on vagal afferents from the NGA plays an obligatory role in controlling alcohol intake, with particular importance for the *Avil* and *Gpr65* co-expressing subset that innervates the mucosal layer of the GI tract. This is supported by several lines of evidence. First, tamoxifen-induced deletion of CB_1_R from *Avil*-expressing neurons in *Avil*^*CreERT2*^;*Cnr1*^*lox/lox*^ mice resulted in a significant reduction in VEI and eliminated the inhibitory effect of JD5037, implying a strong sensory neuronal component and active endocannabinoid tone at the vicinity of neuronal CB_1_R. Even though tamoxifen displays micromolar binding affinity for CB_1_R, it is unlikely that low baseline ethanol intake in *Avil*^CreERT2^;*Cnr1*^*lox/lox*^ mice resulted from a tamoxifen interaction with CB_1_R, as similar treatment of *Avil*^wt^;*Cnr1*^*lox/lox*^ mice did not change baseline VEI and didn’t influence the JD5037’s inhibitory effect. Second, the loss of VEI inhibition by JD5037 occurs only when *Cnr1* is selectively deleted from NGA, but not from DRG neurons. Third, it was unexpected to find *Trpv1*^Cre^;*Cnr1*^*lox/lox*^ mice remaining sensitive to JD5037 in light of our earlier observations showing a loss of sensitivity to CB_1_R and Ghsr antagonists upon capsaicin-induced vagal deafferentation [20]. This discrepancy may arise from a poor overlap of *Cnr1* and *Trpv1* mRNA in NGA and limited selectivity of capsaicin and subdiaphragmatic vagotomy as tools for vagal deafferentation [43]. Alternatively, vagal deafferentation may interrupt Ghsr signaling via capsaicin-sensitive neurons [25], whereas CB_1_R modulates alcohol-related behaviors through a Trpv1^-^ subset of vagal neurons. Fourth, inhibition of VEI by JD5037 was mediated selectively through CB_1_R located on GPR65^+^/Phox2b^+^ vagal sensory neurons that innervate the mucosal layer of the gut, whereas CB_1_R on Glp1r^+^/Phox2b^+^ vagal afferents innervating the muscle layer are not involved. Finally, targeted lesioning of CB_1_R-expressing NGA neurons with CB_1_-saporin was associated with a selective disappearance of neurons expressing both *Cnr1* and *Phox2b* and mirrored the loss of sensitivity to JD5037 observed in Phox2b^Cre^;CB_1_^*lox/lox*^, Gpr65^iresCre^;CB_1_^*lox/lox*^ and Avil^CreERT^;CB_1_^*lox/lox*^ mice, ruling out non-vagal neurons that may aberrantly express Phox2b and advillin. Since alcohol exposure did not change CB_1_R expression or endocannabinoid levels in the NGA, this supports our earlier observations regarding the role of gut-derived endocannabinoids in the modulation of alcohol consumption via pathways involving NGA projections from the gastrointestinal tract [20].

The study explores the extrinsic innervation of the GI tract, focusing on two types of sensory neurons from NGA: Glp1r-expressing neurons sensing gastrointestinal stretch and control feeding [6, 7, 21, 22], and Gpr65^+^ afferents targeting intestinal villi and responding to chemical cues [21]. As a pH-sensitive GPCR sensing acidic pH, Gpr65 has been implicated in the regulation of gut inflammatory responses from nausea-inducing toxins, commensal microflora and inflammatory cues [22, 49, 50], such as ethanol, suggesting a link with immune and inflammatory responses associated with AUD. Our study substantiates the involvement of CB_1_R on Gpr65^+^ NGA neurons in regulating VEI. The direct contact of alcohol with the mucosa that lines the upper GI tract can trigger the production of ECs [20] leading to activation of CB_1_R on Gpr65^+^ afferents, signaling to the CNS to promote alcohol drinking, while peripheral CB_1_R blockade reduces alcohol drinking by breaking the positive reinforcing loop (Graphical Abstract).

The molecular specialization of NGA neurons is complex, with at least 18 clusters of neurons differing in their gene expression profile reflecting the diversity of vagus nerve neurons and sensory functions [9]. *Phox2b* is a common genetic marker, highly expressed in all clusters*. Avil* is expressed in most clusters except for N6, N8 and N13, while *Trpv1* expression overlaps with the cholecystokinin receptor (*Cckr*), highly enriched in cluster N13, moderately in clusters N6-16 and poorly in others [9]. Notably, there is a poor overlay among clusters expressing *Cnr1* and *Trpv1*, whereas *Avil* and *Cnr1* highly overlap in clusters 17 and 18, explaining reduced drinking and loss of JD5037 effect in mice with selective *Cnr1* deletion in *Avil-*expressing but not *Trpv1-*expressing NGA neurons. Consistently, *Gpr65* expression is restricted to cluster N17 along with *Avil* and, to a lesser extent, cluster N18, neither of which expresses *Trpv1* or *Cckr* [9]. *Gpr65* expression is uniquely localized in NGA neurons innervating the mucosal layer of the GI tract where neuroendocrine cells, including ghrelin-producing cells of the stomach, reside. They do not project to the lung or have a direct known cardiopulmonary role [21]. The gut also contains an intrinsic network of myenteric neurons, which express *Cnr1* but not *Gpr65* [51]. Thus, it is unlikely that CB_1_R in myenteric neurons contributes to VEI control.

A recent study revealed asymmetric ascending vagal pathways in the CNS responsible for gut-induced reward [8]. Vagal afferents from the right nodose ganglion activate a parabrachio-nigral pathway mediating reward-related behaviors, while a different subset likely controls satiety and aversive behaviors. Surprisingly, we found higher *Cnr1* expression in the right vs. left NGA and more pronounced ablation of CB_1_R-expressing cell bodies in the right NGA of vagal afferents by CB1 SAP, suggesting asymmetry in controlling VEI by CB1R antagonists. However, limitations of our model prevent conclusive statements on unilateral engagement of the right NGA. Further research is needed to fully understand the role of CB_1_R in modulating these asymmetrical vagal pathways and their implications for the regulation of alcohol intake.

The pharmacological specificity of JD5037 for peripheral neuronal CB_1_R is reinforced here by another peripherally restricted CB_1_R inhibitor, MRI-1891, which reproduced the effects of JD5037 in inhibiting VEI in two different models in male and female mice. Furthermore, the CB_1_R specificity of MRI-1891 is indicated by its inhibitory effect residing in its *S* enantiomer, which has sub-nanomolar binding affinity to CB_1_R, not in its *R* enantiomer which has 3 orders of magnitude lower affinity for CB_1_R.

A seminal study by Date and colleagues detected *Ghsr* mRNA in the rat NGA using PCR and in situ hybridization [25]. Subsequent studies corroborated *Ghsr* expression in the rat NGA [52] and reported Ghsr-immunoreactive cells expressing the cholecystokinin receptor [53]. The extent of Ghsr expression in mouse vagal sensory neurons is controversial. Some studies detected *Ghsr* mRNA in the mouse NGA [52, 54], while others did not [9, 11]. *Ghsr* mRNA was found in mouse NGA neurons innervating the stomach that also expressed *Glp1r*, *Trpv1* and *Cnr1* [54]. Ghrelin was shown to hyperpolarize cultured NGA cells [54], abolish CCK-induced expression of cocaine- and amphetamine-regulated transcript [55], a neurohormone regulating food intake and reward and affecting alcohol drinking behavior [56]. Our earlier experiments hinted at the possible involvement of vagal sensory afferent Ghsr in controlling alcohol drinking, although experimental conditions limited more specific evidence [20].

This study supports *Ghsr* mRNA expression in a discrete population of Phox2b^+^ mouse NGA neurons, as detected using RNAscope. A functional role for Ghsr in the control of VEI is suggested by selective Ghsr deletion in *Phox2b*^Cre^;*Ghsr*^*lox/lox*^ transgenic mice, resulting in reduced baseline VEI and loss of sensitivity to the peripheral Ghsr antagonist PF-5190457. These animals also failed to respond to JD5037, whereas *Phox2b*^Cre/wt^; *Cnr1*^*lox/lox*^ animals were no longer sensitive to PF-5190457. Thus, our findings suggest that CB_1_R and Ghsr play an interdependent role in regulating VEI. CB_1_R and Ghsr can crosstalk directly on the same neurons or interact through converging signaling pathways in the brainstem. Specifically, CB_1_R may regulate the activity of Gpr65^+^ NGA neurons that are capsaicin-insensitive, while Ghsr may be on capsaicin-sensitive Trpv1^+^ neurons responding to ghrelin. Further studies are warranted to explore the nature of the interaction of CB_1_R and Ghsr in the control of VEI.

The gut and brain communicate bidirectionally through the vagus nerve to regulate diverse physiological functions, such as digestion, respiration, blood pressure, heart rate, mood and immune responses [40, 57]. The importance of this gut-brain axis is illustrated by mutations in Phox2b, which lead to a wide range of pathological conditions e.g., gastroparesis, heart failure, impaired respiratory control [21, 40]. It also plays a key role in translating gut-derived signals, especially those triggered by palatable foods, into neural activity that influences appetite and food-seeking behavior. It remains to be determined whether CB_1_R in a specific subpopulation of NGA controls not only VEI but also reward-driven palatable food preferences or other functions. The tone of the vagus nerve is controlled, among others, by ECB and ghrelin, affecting alcohol drinking, as evidenced by the change in the drinking behavior in mice lacking the CB_1_R or Ghsr receptor in a population of Phox2b^+^ or Avil^+^ NGA neurons. The modulation of vagal tone by CB_1_R and Ghsr is also supported by previous studies [22, 25, 58]. Non-invasive vagus nerve stimulation (nVNS) shows promise as an adjunct treatment for substance use disorders and addiction. Animal and human studies show that nVNS can reduce drug seeking, reinforce abstinence, and improve depression and sleep quality in alcohol-dependent patients after withdrawal [59]. At least one ongoing clinical trial investigates nVNS potential in reducing relapse rates and improving functional outcomes in AUD patients (https://www.clinicaltrials.gov/ct2/show/NCT05226130). Another study looks at the association between alcohol, the vagus nerve and multi-organ inflammation (https://grantome.com/grant/NIH/R21-AA020188-01). This study identifies a specific subset of neurons that may be targeted by nVNS to treat AUD. The proximity of these neurons to the gut epithelium is crucial given the growing evidence suggesting that alcohol addiction may originate in the gut or increase susceptibility to substance use disorders [60]. Since innervation of the gut mucosal layer appears to be critical in modulating VEI, developing a gut-restricted CB_1_R antagonist or small-molecule inhibitor targeting Gpr65 could be an efficacious approach with limited side effects compared to global or non-brain penetrant molecules. Finally, our findings provide a plausible mechanism underlying the therapeutic effectiveness in AUD of non-brain penetrant CB_1_R antagonists, including MRI-1891 [32]. In a recent phase 1b clinical trial, MRI-1891 (renamed as INV-202 or monlunabant) showed high anti-obesity efficacy in people with the metabolic syndrome [61] and is currently in a phase 2 clinical study. The present findings strongly warrant the clinical testing of MRI-1891/ INV-202 for treating AUD.

In conclusion, our study provides a new link between peripheral CB_1_R and the central regulation of VEI. It highlights the importance of CB_1_R receptors on subsets of NGA neurons co-expressing Gpr65 and advillin in modulating alcohol drinking behavior via afferent neuronal signaling and provides a functional basis for the testing peripheral CB_1_R antagonists in treating AUD. Importantly, our findings support the potential of this approach in both female and male subjects.

## Supplementary information

Supplemental Material

Data source

## Data Availability

This study does not contain any omics data, which are required to be deposited in a public repository. All data that support the findings of this study are available within the article, its Supplementary Information or Source Data file, which is provided with this paper. Original RNAscope images and data sets supporting the Source Data file are accessible in the Zenodo open online data repository (10.5281/zenodo.16990200, 10.5281/zenodo.16969126, 10.5281/zenodo.16968162, 10.5281/zenodo.16950891, 10.5281/zenodo.16996512). PCR gels are shown uncropped in Figure S1. Ghsr conditional knockout mice and any other data, will be provided upon request for research purpose only.
