# Supplementary material for: CB1 receptors on a subset of vagal afferent neurons modulate voluntary ethanol intake in mice
Source: Mol Psychiatry. 2025 Sep 20;31(1):48–61. doi: 10.1038/s41380-025-03266-9 (PMC12700788; doi:10.1038/s41380-025-03266-9)
Supplement: Supplementary file 1 — Supplemental Material [file 41380_2025_3266_MOESM1_ESM.pdf]

CB_1 receptors on a subset of vagal afferent neurons modulate voluntary ethanol intake in mice

Alexa Herrerias[1], Anna Oliverio[1], Szabolcs Dvorácskó, PhD[2,3], Arthi Thyagarajan[1], Lee Chedester, DVM[1], Jie Liu, PhD[1], Resat Cinar, PhD [2], Malliga R. Iyer, PhD[3], George Kunos, PhD[1], Grzegorz Godlewski, PhD[1].

[1]Laboratory of Physiologic Studies, Division of Intramural Clinical and Biological Research, National Institute on Alcohol Abuse and Alcoholism, National Institutes of Health (NIH), Bethesda, MD 20892, USA.

[2]Section on Fibrotic Disorders, Division of Intramural Clinical and Biological Research, National Institute on Alcohol Abuse and Alcoholism, National Institutes of Health (NIH), Bethesda, MD 20892, USA.

[3]Section on Medicinal Chemistry, Division of Intramural Clinical and Biological Research, National Institute on Alcohol Abuse and Alcoholism, National Institutes of Health (NIH), Bethesda, MD 20892, USA.

Correspondence:

Grzegorz Godlewski
greg.godlewski@nih.gov

George Kunos
george.kunos@nih.gov

Running title: CB_1 receptor and ethanol

Materials and methods

Animals

The following mouse strains were purchased from the Jackson Laboratory (USA): C57BL/6J, *Wnt1-Cre* (B6.Cg-E2f1$^{\text{Tg(Wnt1-cre)2Sor}}$/J), *Phox2b-Cre* (B6(Cg)-Tg(Phox2b-cre)3Jke/J), *Trpv1-Cre* (B6.129-Trpv1$^{\text{tm1(cre)Bbm}}$/J), Advillin-CreERT2 (Tg(Avil-icre/ERT2)AJwo/J). Gpr65^{iresCre} and Glp1r$^{\text{ires-Cre}}$ strains were kindly provided by Dr. Stephen Liberles [1,2] (Harvard Medical School, Boston, USA), the ghrelin-Cre knock-in mouse line by Dr. Michael Mulholland and Dr. Randy Seeley [3] (University of Michigan, USA). *Cnr1*$^{lox/lox}$ was from Dr Josephine M. Egan [4] (National Institute on Aging, NIH), *Cnr1*$^{-/-}$ from Dr Zimmer [5] (National Institute of Mental Health, NIH).

To achieve the desired tissue-specific receptor deletion, Cre expressing mice were bred with *Cnr1*$^{lox/lox}$ or *Ghsr*$^{lox/lox}$ mice. All other strains were propagated by heterozygote breeding.

Generation of *Ghsr* conditional knockout mice

The conditional knockout *Ghsr*$^{\text{lox/lox}}$ mouse line was generated using CRISPR/Cas9-mediated genome engineering by Cyagen Biosciences Inc.-Taconic (USA) on a contract with our laboratory (contract number: NTMCC-190507-ACD-01).

The three exons of the *Ghsr* gene (NCBI reference sequence: NM_177330; Ensembl: ENSMUSG00000051136, transcript: ENSMUST00000057186) were identified on mouse chromosome 3, with the ATG start codon in exon 2 and TGA stop codon in exon 3. Exon 2 was selected as conditional knockout region since the deletion of this region was expected to result in the loss of function of the *Ghsr* gene. Mouse genomic fragments containing homology arms were amplified from BAC by using the high fidelity Taq DNA polymerase and were sequentially assembled into a targeting vector together with recombination sites. The donor vector was designed to insert a loxP cassette 5' 7 bp upstream of the ATG start codon of the mouse *Ghsr*

gene on exon 2 and a 3' loxP-FRT-Neomycin-FRT (Neo) cassette 1200 bp down, outside exon 2. The vector was co-injected into fertilized eggs of C57Bl/6J mice along with the endonuclease Cas9 mRNA, and the short guide RNA target sequences to mouse Ghsr1a gene (gRNA1, matching reverse strand of gene: GTCGCGTTCCACATGTTGCCAGG, gRNA2, matching forward strand of gene: GTGTAGTAAAATCACGAAGACGG). The vector contained 1259 bp of the complete coding sequence of the murine *Ghsr* gene in exon 2 followed by 1200 bp intron sequence in the homologous arms. Cre recombination deleted entire coding region in exon2 with partial sequence remaining in exon 3 in the knock-out mice. The F0 founder offspring animals were identified by the PCR followed by DNA sequence analysis, which were bred to wild-type mice to test germline transmission and F1 animal generation. The founders were genotyped by PCR followed by DNA sequencing analysis, confirming a 3.2 kb insertion. The positive founders were bred to the next generation (F1) and subsequently genotyped by PCR and DNA sequencing analysis. Animals were on a C57BL/6J background.

Genotyping and primer sequences

The following forward (F) and reverse (R) primer sequences were used:

Ghrl$^{\mathbf{Cre}}$: F1 common (5'-TAGAAGACTGACTTGATCTCTTGC-3') and R1 control (5'-TGAACATTTGGCATACAGGTGAAC-3') and R2 transgene (5'-CGATCCCTGAACATGTCCATCAG-3') amplified 183 bp and 215 bp segments in Cre negative and positive mice, respectively.

Avil$^{\mathbf{Cre}}$: F1 common (5'-CCCTGTTCACTGTGAGTAGG-3') and R1 control (5'-AGTATCTGGTAGGTGCTTCCAG-3') and R2 transgene (5'-GCGATCCCTGAACATGTCCATC-3') amplified 500 bp and 180 bp segments in Cre negative and positive mice, respectively.

Trpv1$^{\mathbf{Cre}}$: F1 control (5'-TTCAGGGAGAAACTGGAAGAA-3') and R1 control (5'-TAGTCCCAGCCATCCAAAAG-3') and F2 transgene (5'-GCGGTCTGGCAGTAAAAACTATC)

and R2 transgene (5'-GTGAAACAGCATTGCTGTCACTT-3') amplified 102 bp and 490 bp segments in Cre negative and positive mice, respectively.

Phox2b^{Cre}: F1 common (5'-CCGTCTCCACATCCATCTTT-3') and R1 control (5'-GTACGGACTGCTCTGGTGGT-3') and R2 transgene (5'-ATTCTCCCACCGTCAGTACG-3') amplified 300 bp and 700 bp segments in Cre negative and positive mice, respectively.

Wnt1^{Cre}: F1 control (5'-CAAATGTTGCTTGTCTGGTG-3') and R1 control (5'-GTCAGTCGAGTGCACAGTTT-3') and also F2 transgene (5'-CAGCGCCGCAACTATAAGAG-3') and R2 transgene (5'-CATCGACCGGTAATGCAG-3') amplified 200 bp and 475 bp segments in Cre negative and positive mice, respectively.

Glp1r^{iresCre}: F1 common (5'-GTTTCTTCCTCTCTTCCTGCC-3') and R1 control (5'-TCATCAAGCCCATCTCTCTCC-3') and R2 transgene (5'-ACCGCTTCCTCGTGCTTTAC-3') amplified 128 bp and 80 bp segments in Cre negative and positive mice, respectively.

Gpr65^{iresCre}: F1 control (5'-GGAGAGCTGATATGTGGAAC-3') and R1 control (5'-CCAGTACATGGAGTGGCTTC-3') and F2 transgene (5'-CCTGTTTCGGACGTGCATGG-3') and R2 transgene (5'-GGAGGGAGAGGGGCGGAATT-3') amplified 555 bp and 480 bp segments in Cre negative and positive mice, respectively.

Cnr1$^{lox/lox}$: F common (5'-GGGCCCTTTTCCAATAAAGAGAAGTATTGC-3') and R common (5'-CAAGAGACTGCAGCAGGCCAAATCTAG-3') amplified 255 bp and 367 bp segments of the wild type and mutant alleles, respectively.

Ghsr1$^{lox/lox}$: F common (5'-TTGTCCTTCAGGAAGCTCTGGATA-3') and R common (5'-CCTTGAAAAGTGAGATCAGTGGTT-3') amplified 155 bp and 228 bp segments of the wild type and mutant alleles, respectively.

Cnr1: F1 wild type (5'-GTACCATCACCACAGACCTCCTC-3') and R1 wild type (5'-GGATTCAGAATCATGAAGCACTCCA-3') and R2 mutant (5'-AAGAACGAGATCAGCAGCCTCTGTT-3') amplified a 350 bp and 150 bp segments of the gene in wild type and mutant mouse, respectively.

Fluorescent *in situ* hybridization probes

The following mouse RNAscope® FISH probes from ACD were used: *Cnr1* in channel 2 (NM_007726.3, region 701 – 1792; reference number 457341-C2), *Phox2B* in channels 1 and 3 (NM_008888.3, region 1617 – 2790; reference numbers 407861 and 407861-C3), *Ghrl* in channel 2 (NM_021488.5, region 13 – 550; reference number 415301-C2), *Trpv1* in channels 1 and 3 (NM_001001445.1, region 1162 – 2155; reference numbers 313331 and 313331-C3), *Glp1r* in channel 2 (NM_021332.2, region 108-1203; reference number 418851-C2), *Gpr65* in channel 2 (NM_008152.3, region 521 – 1652; reference number 431431-C2), and *Avil* in channel 2 (NM_009635.3, region 354 – 1322; reference number 498531-C2). To assess of the effectiveness of gene deletion in tissues obtained from *Cnr1*$^{lox/lox}$ or *Ghsr1a*$^{lox/lox}$ mice, the respective probes *were* designed to recognize floxed alleles: *Cnr1-O1* in channels 1 and 2 (NM_007726.3, region 701 – 1792; reference numbers 457341 and 457341-C2) and *Ghsr-O2* in channel 1 (NM_177330.4, region 270 – 1025; reference number 1147851-C1).

TSA Vivid™ Fluorophores 520, 570 and 650 (Tocris Bioscience, USA) were used to visualize the RNAscope® FISH probes. Probe dilutions ranged from 1:750 to 1:1500, except for *Ghsr-O2* (1:500) and *Ghrl* (1:40000). DAPI was used to stain for nuclei.

Graphical abstract

Graphical abstract was prepared with the scientific image and illustration software BioRender (www.BioRender.com, USA).

References

1. Chang RB, Strochlic DE, Williams EK, Umans BD, Liberles SD. Vagal Sensory Neuron Subtypes that Differentially Control Breathing. Cell. 2015;161(3):622-33.
2. Williams EK, Chang RB, Strochlic DE, Umans BD, Lowell BB, Liberles SD. Sensory Neurons that Detect Stretch and Nutrients in the Digestive System. Cell. 2016;166(1):209-21.
3. Engelstoft MS, Park WM, Sakata I, Kristensen LV, Husted AS, Osborne-Lawrence S, et al. Seven transmembrane G protein-coupled receptor repertoire of gastric ghrelin cells. Mol Metab. 2013;2(4):376-92.
4. Gonzalez-Mariscal I, Montoro RA, Doyle ME, Liu QR, Rouse M, O'Connell JF, et al. Absence of cannabinoid 1 receptor in beta cells protects against high-fat/high-sugar diet-induced beta cell dysfunction and inflammation in murine islets. Diabetologia. 2018;61(6):1470-83.
5. Zimmer A, Zimmer AM, Hohmann AG, Herkenham M, Bonner TI. Increased mortality, hypoactivity, and hypoalgesia in cannabinoid CB1 receptor knockout mice. Proc Natl Acad Sci U S A. 1999;96(10):5780-5.

Supplementary Figure Legends

Figure S1. Representative genotyping results of transgenic mouse strains by the reverse transcription-PCR.

Upper gel sections show bands from 3-4 transgenic mice with negative (control; ctrl) and positive (Cre) recombinase, driven by the following promoter genes: *Ghrl* (A), *Avil* (B), *Trpv1* (C), *Phox2b* (D), *Wnt1* (E), *Glp1r* (F), *Gpr65* (G) and *Phox2b* (H). Lower sections show *Cnr1*$^{lox/lox}$ (A-G) and *Ghsr*$^{lox/lox}$ (H). The last sample comes from C57BL/6J mouse, which represents Cre-negative, non-floxed specimen.

Figure S2. Effect of ablation of CB_1R-expressing NGA neurons via saporin-CB_1R antibody conjugates on VEI.

Cnr1 and *Phox2b* expression in NGA tissue sections after bilateral intraganglion injection of blank saporin (blank) or CB1-saporin (CB1 SAP) as visualized by fluorescent RNAscope imaging. Co-localization was assessed by overlaying 2 channels. Arrows show examples of the co-localization of mRNA for CB_1R (red) and Phox2b (green). DAPI (blue) visualizes nuclear DNA.

Figure S3. Non-brain penetrant CB_1R antagonists regulate VEI through peripheral sensory neuronal CB_1R in female mice.

(A) The effect of oral administration of JD5037 or vehicle on VEI in and CB_1R-deficient (*Cnr1*$^{-/-}$) and wild-type (wt) littermate female mice in the two-bottle choice test.

(B) The effect of CB_1R blockade with *S* and *R* enantiomers of MRI-1891 or vehicle (veh) on VEI in control (ctrl) (*Phox2b*^{wt};*Cnr1*$^{lox/lox}$) and nodose ganglion-specific CB_1R-deficient (Cre) (*Phx2b*^{Cre};*Cnr1*$^{lox/lox}$) littermate mice in the drinking-in-the-dark paradigm.

(C) The corresponding serum ethanol in in control (ctrl) (*Phox2b*^{wt};*Cnr1*$^{lox/lox}$) and nodose ganglion-specific CB_1R-deficient (Cre) (*Phx2b*^{Cre}; *Cnr1*$^{lox/lox}$) littermates subjected to the drinking-in-the dark paradigm and treated with *S* and *R* enantiomers of MRI-1891 (MRI) or vehicle (V).

Points and bars represent means ± SEM from the indicated number of experiments (A-C). Drinking behavior in individual animals is expressed as points before (average of days 1–3) and after treatment on day 4 (B). *$P < 0.05$, **$P < 0.01$ by two-way ANOVA (A), Student's t-test for paired samples (B), or by Kruskal–Wallis test (C, left bar graph). Raw data points and the associated estimates of variation are accessible in the source data file.

Figure S4. *Gpr65* is co-expressed with *Avil* on the same subset of NGA neurons

Gpr65 and`*Avil* as visualized by fluorescent RNAscope imaging of NGA tissue sections from C57BL6/J mice. Co-localization was assessed by overlaying 2 channels. Arrows are examples showing overlapping mRNA for *Gpr65* (green) and advillin (*Avil*) (red). DAPI (blue) visualizes nuclear DNA. Yellow square indicates the area displayed at 20x magnification. Adjacent bar graphs indicate the average expression of *Avil* in *Gpr65*-positive NGA cells in all samples at 20x magnification.

Bars represent means ± SEM from the indicated number of experiments. Raw data points and the associated estimates of variation are accessible in the source data file.

Figure S5. Effect of alcohol drinking on *Cnr1* expression and endocannabinoid levels in alcohol drinking mice.

(A) *Cnr1* (red) and *Phox2b* (green) mRNA expression as visualized by fluorescent RNAscope imaging of NGA tissue sections from alcohol-naïve (control) and alcohol drinking male C57BL/6J mice subjected go the two bottle-choice test and drinking-in the dark paradigms. Yellow squares in images taken with 10x microscope objective (left column) indicate areas displayed at 20x magnification. Co-localization of *Cnr1* (red) and *Phox2b* (green) mRNA was presented by overlaying 2 channels. DAPI (blue) visualizes nuclear DNA. Adjacent bar graphs indicate the average intensity of fluorescence of red and green fluorophores from all samples at 20x magnification.

(B) *Cnr1* gene expression by RT-qPCR in NGA tissues from alcohol-naïve (control) and alcohol drinking male C57BL/6J mice subjected go the two bottle-choice test and drinking-in the dark paradigms, as described in (A).

(C) Endocannabinoid levels (anandamide, AEA; 2-arachidonoyl glycerol, 2-AG) measured in NGA from alcohol-naïve (control) and alcohol drinking male C57BL/6J mice subjected go the two bottle-choice test and drinking-in the dark paradigms, as described in (A).

(D) Oleoylethanolamide (OEA) levels measured in different tissues from alcohol-naïve (control) and alcohol-exposed male C57BL/6J mice during the two-bottle choice test described in (A).

Bars represent means ± SEM from the indicated number of experiments. **P < 0.01 by Student's t-test. Raw data points and the associated estimates of variation are accessible in the source data file.

Figure S1

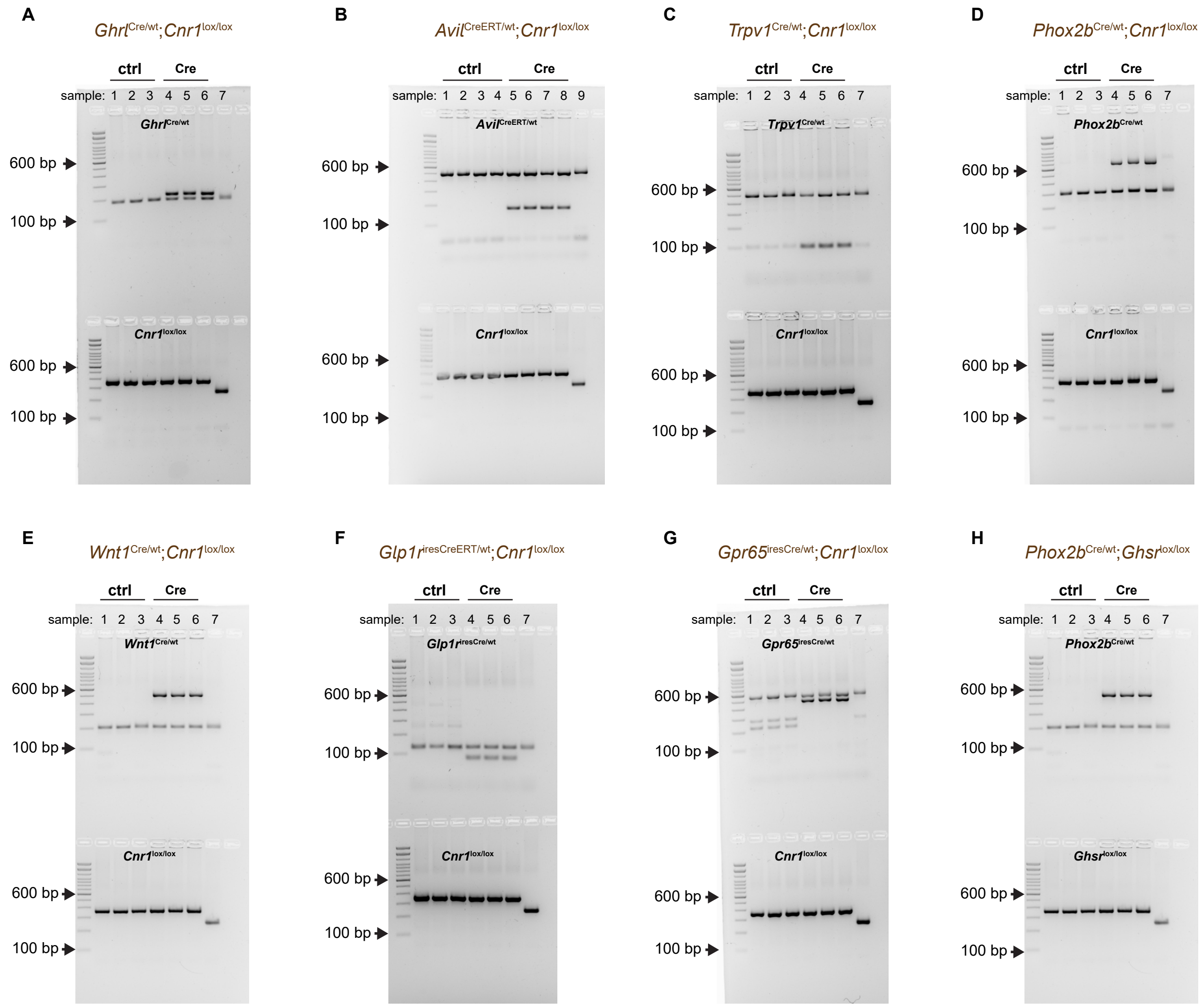
A
$Ghrl^{Cre/wt}$;$Cnr1^{lox/lox}$
ctrl
Cre
sample: 1 2 3 4 5 6 7
$Ghrl^{Cre/wt}$
600 bp
100 bp
$Cnr1^{lox/lox}$
600 bp
100 bp
B
$Avil^{CreERT/wt}$;$Cnr1^{lox/lox}$
ctrl
Cre
sample: 1 2 3 4 5 6 7 8 9
$Avil^{CreERT/wt}$
600 bp
100 bp
$Cnr1^{lox/lox}$
600 bp
100 bp
C
$Trpv1^{Cre/wt}$;$Cnr1^{lox/lox}$
ctrl
Cre
sample: 1 2 3 4 5 6 7
$Trpv1^{Cre/wt}$
600 bp
100 bp
$Cnr1^{lox/lox}$
600 bp
100 bp
D
$Phox2b^{Cre/wt}$;$Cnr1^{lox/lox}$
ctrl
Cre
sample: 1 2 3 4 5 6 7
$Phox2b^{Cre/wt}$
600 bp
100 bp
$Cnr1^{lox/lox}$
600 bp
100 bp
E
$Wnt1^{Cre/wt}$;$Cnr1^{lox/lox}$
ctrl
Cre
sample: 1 2 3 4 5 6 7
$Wnt1^{Cre/wt}$
600 bp
100 bp
$Cnr1^{lox/lox}$
600 bp
100 bp
F
$Glp1r^{iresCreERT/wt}$;$Cnr1^{lox/lox}$
ctrl
Cre
sample: 1 2 3 4 5 6 7
$Glp1r^{iresCre/wt}$
600 bp
100 bp
$Cnr1^{lox/lox}$
600 bp
100 bp
G
$Gpr65^{iresCre/wt}$;$Cnr1^{lox/lox}$
ctrl
Cre
sample: 1 2 3 4 5 6 7
$Gpr65^{iresCre/wt}$
600 bp
100 bp
$Cnr1^{lox/lox}$
600 bp
100 bp
H
$Phox2b^{Cre/wt}$;$Ghsr^{lox/lox}$
ctrl
Cre
sample: 1 2 3 4 5 6 7
$Phox2b^{Cre/wt}$
600 bp
100 bp
$Ghsr^{lox/lox}$
600 bp
100 bp

Figure S2

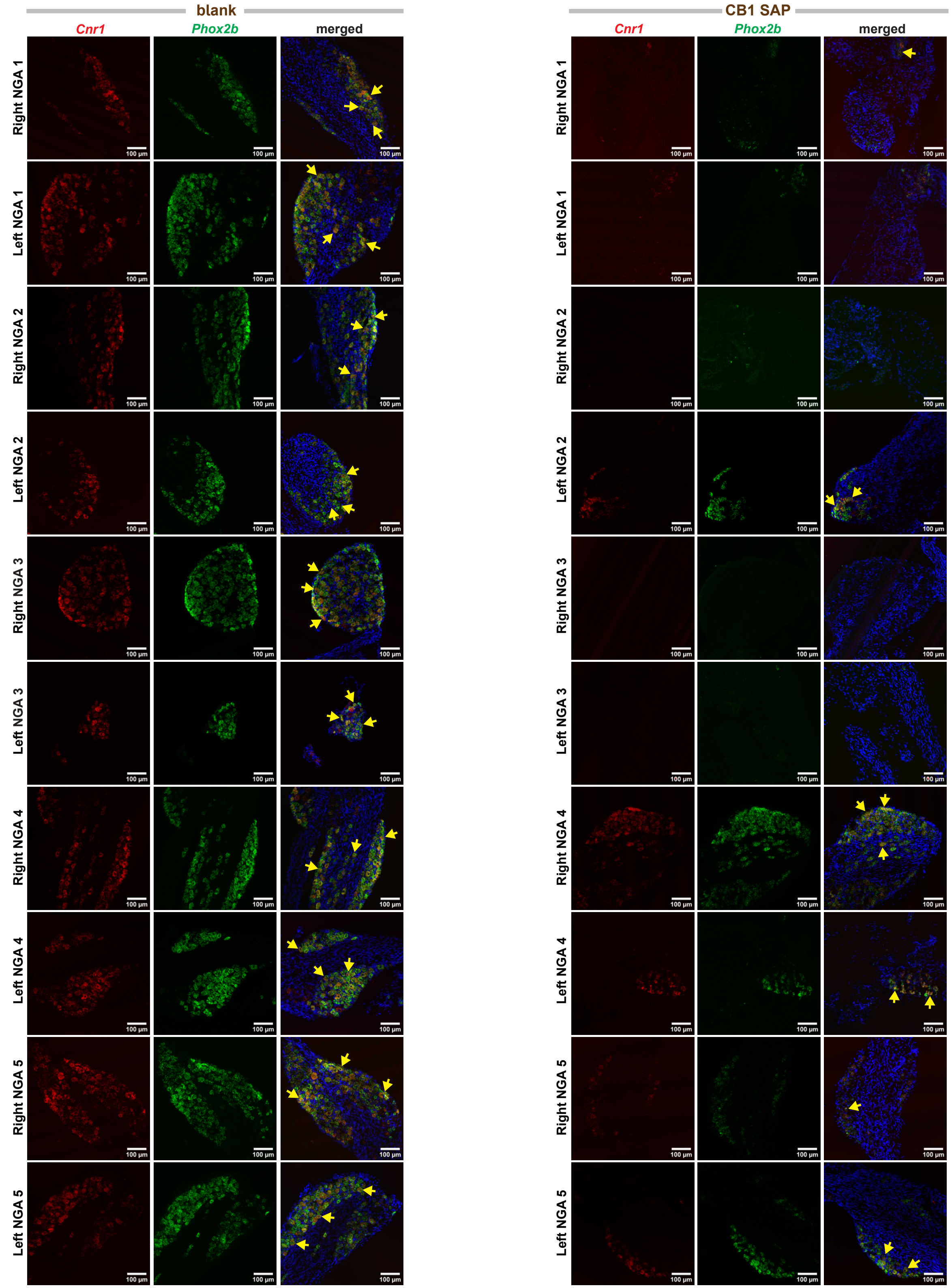
blank
CB1 SAP
Cnr1
Phox2b
merged
Right NGA 1
Left NGA 1
Right NGA 2
Left NGA 2
Right NGA 3
Left NGA 3
Right NGA 4
Left NGA 4
Right NGA 5
Left NGA 5
100 µm

Figure S3

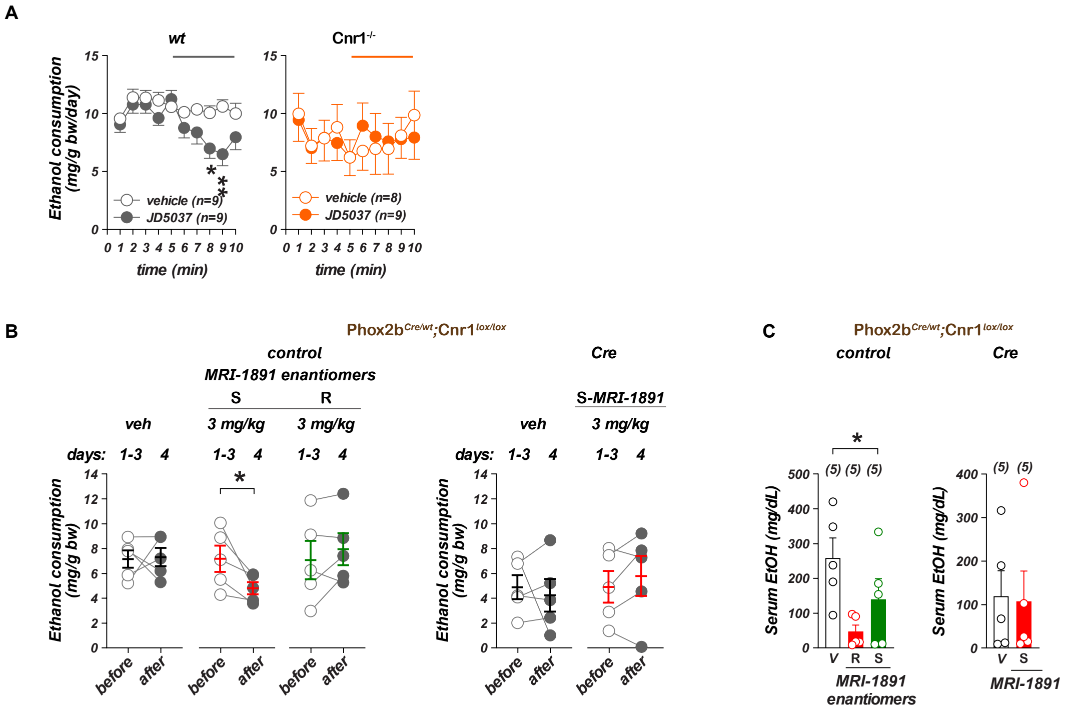
A
wt
Cnr1-/-
Ethanol consumption (mg/g bw/day)
vehicle (n=9)
JD5037 (n=9)
vehicle (n=8)
JD5037 (n=9)
time (min)
B
Phox2bCre/wt;Cnr1lox/lox
control
MRI-1891 enantiomers
S
R
veh
3 mg/kg
days: 1-3 4
Ethanol consumption (mg/g bw)
before
after
Cre
S-MRI-1891
C
Serum EtOH (mg/dL)
(5)
V
R
S
MRI-1891 enantiomers
MRI-1891

Figure S4

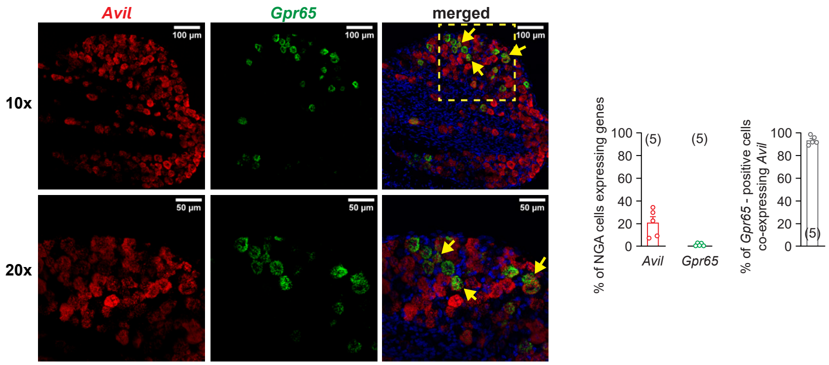

Avil
Gpr65
merged
10x
20x
100 μm
50 μm
% of NGA cells expressing genes
100
80
60
40
20
0
(5)
(5)
Avil
Gpr65
% of Gpr65 - positive cells co-expressing Avil
(5)

Figure S5

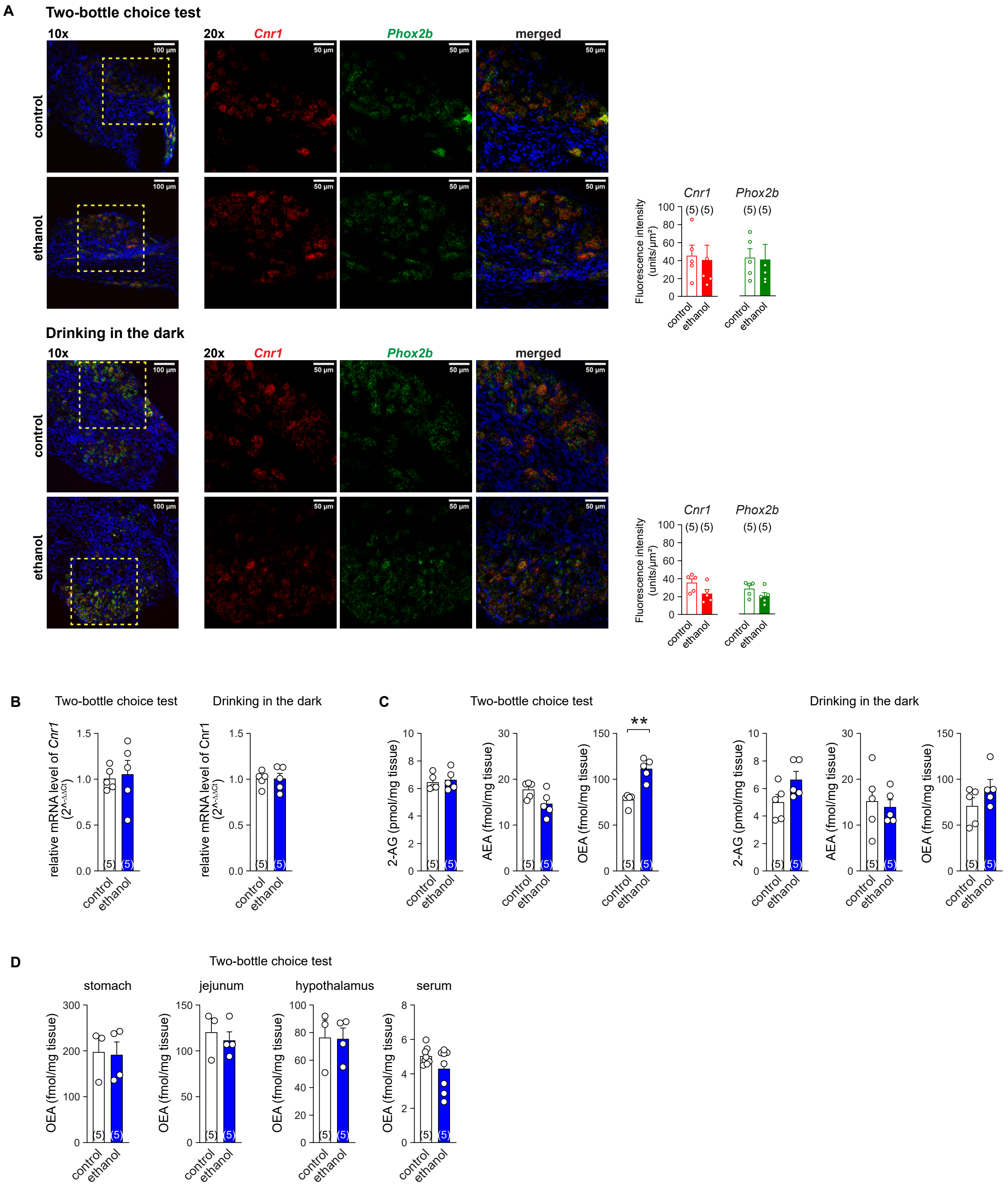
A
Two-bottle choice test
10x
20x
Cnr1
Phox2b
merged
control
ethanol
100 µm
50 µm
Cnr1
Phox2b
(5) (5)
Fluorescence intensity (units/µm²)
100
80
60
40
20
0
control
ethanol
Drinking in the dark
B
Two-bottle choice test
Drinking in the dark
relative mRNA level of Cnr1 (2^-ΔΔCt)
1.5
1.0
0.5
0.0
C
Two-bottle choice test
Drinking in the dark
2-AG (pmol/mg tissue)
AEA (fmol/mg tissue)
OEA (fmol/mg tissue)
**
D
Two-bottle choice test
stomach
jejunum
hypothalamus
serum
OEA (fmol/mg tissue)
(5)

Table S1. Baseline daily consumption of ethanol and total liquid (ethanol + water) in mice on a two-bottle/free choice test.

**Transgene**	**Treatment**	**Ethanol consumption (mg/g bw/day)**	**Total drinking (µL/g bw/day)**	**N**
Ghrl^{wt};*Cnr1*$^{lox/lox}$		11.85 ± 0.67	129.04 ± 4.81	23
Ghrl^{Cre};*Cnr1*$^{lox/lox}$		10.33 ± 0.63	126.43 ± 3.51	37
Avil^{wt};*Cnr1*$^{lox/lox}$	corn oil	12.84 ± 0.52	129.15 ± 2.81	47
Avil^{CreERT};*Cnr1*$^{lox/lox}$	corn oil	12.32 ± 0.74	119.70 ± 5.86	23
Avil^{wt};*Cnr1*$^{lox/lox}$	tamoxifen	13.72 ± 0.60 ***	131.62 ± 3.18	58
Avil^{CreERT};*Cnr1*$^{lox/lox}$	tamoxifen	7.52 ± 0.75	131.89 ± 3.35	70
Trpv1^{wt};*Cnr1*$^{lox/lox}$		13.56 ± 0.53	128.16 ± 2.98	33
Trpv1^{Cre};*Cnr1*$^{lox/lox}$		12.93 ± 0.52	123.25 ± 2.47	51
C57BL/6J		10.19 ± 0.32	108.32 ± 2.26	37
C57BL/6J	blank saporin	10.84 ± 0.61	128.61 ± 3.23	12
C57BL/6J	CB1 saporin	11.29 ± 0.86	121.95 ± 3.50	12
Cnr1$^{+/+}$ female		10.38 ± 0.34	156.20 ± 6.20	16
Cnr1$^{-/-}$ female		8.18 ± 0.97 *	152.27 ± 4.14	17
Phox2b$^{/wt}$;*Cnr1*$^{lox/lox}$		11.13 ± 0.46 *	128.02 ± 3.66	58
Phox2b^{Cre};*Cnr1*$^{lox/lox}$		8.77 ± 0.46	121.41 ± 3.485	71
Wnt1^{wt};*Cnr1*$^{lox/lox}$		11.59 ± 0.65	127.72 ± 4.37	24
Wnt1^{Cre};*Cnr1*$^{lox/lox}$		11.67 ± 0.64	137.82 ± 4.45	29
Glp1r^{wt};*Cnr1*$^{lox/lox}$		12.55 ± 0.63	141.58 ± 4.73	20
Glp1r^{iresCre};*Cnr1*$^{lox/lox}$		13.14 ± 0.86	144.59 ± 5.11	21

Glp1r^{iresCre}*;Cnr1*$^{lox/lox}$	vehicle	148.42 ± 6.16	11
Glp1r^{iresCre}*;Cnr1*$^{lox/lox}$	JD5037	152.82 ± 5.04	10
Gpr65^{wt}*;Cnr1*$^{lox/lox}$	vehicle	153.31 ± 5.54	14
Gpr65^{wt}*;Cnr1*$^{lox/lox}$	JD5037	165.54 ± 9.02	13
Gpr65^{iresCre}*;Cnr1*$^{lox/lox}$	vehicle	139.13 ± 7.04	10
Gpr65^{iresCre}*;Cnr1*$^{lox/lox}$	JD5037	161.86 ±12.96	12
Phox2b^{wt}*;Ghsr*$^{lox/lox}$	vehicle	150.13 ±16.25	11
Phox2b^{wt}*;Ghsr*$^{lox/lox}$	JD5037	162.16 ±23.47	10
Phox2b^{Cre}*;Ghsr*$^{lox/lox}$	vehicle	173.14 ±21.86	12
Phox2b^{Cre}*;Ghsr*$^{lox/lox}$	JD5037	149.48 ±14.93	13
Phox2b^{wt}*;Ghsr*$^{lox/lox}$	vehicle	150.27 ± 8.40	15
Phox2b^{wt}*;Ghsr*$^{lox/lox}$	PF-05190457	145.68 ± 7.73	15
Phox2b^{Cre}*;Ghsr*$^{lox/lox}$	vehicle	158.29 ±16.36	12
Phox2b^{Cre}*;Ghsr*$^{lox/lox}$	PF-05190457	133.73 ± 5.00	13

Values are means ± SEM from the indicated number (N) of experiments. Where appropriate, the Student's t-test for unpaired samples, Welch's t-test or Mann Whitney U test was used. No statistical differences were detected. A detailed description of the statistical tests used in the study can be found in the Materials and Methods section. Raw data points and the associated estimates of variation are accessible in the source data file.

Table S2. Baseline daily consumption of regular chow in mice undergoing a two-bottle/free choice test.

Transgene	Pretreatment	Drug treatment	Food intake (mg/g bw/day)	N
Ghrl^{wt};*Cnr1*$^{lox/lox}$		vehicle	132.26 ± 5.50	12
Ghrl^{wt};*Cnr1*$^{lox/lox}$		JD5037	147.80 ± 1.26	11
Ghrl^{Cre};*Cnr1*$^{lox/lox}$		vehicle	138.89 ± 3.62	18
Ghrl^{Cre};*Cnr1*$^{lox/lox}$		JD5037	142.50 ± 8.91	19
Avil^{wt};*Cnr1*$^{lox/lox}$	corn oil	vehicle	147.17 ± 4.67	19
Avil^{wt};*Cnr1*$^{lox/lox}$	corn oil	JD5037	153.14 ± 8.29	24
Avil^{CreERT};*Cnr1*$^{lox/lox}$	corn oil	vehicle	145.07 ± 9.09	10
Avil^{CreERT};*Cnr1*$^{lox/lox}$	corn oil	JD5037	145.10 ± 5.64	11
Avil^{wt};*Cnr1*$^{lox/lox}$	tamoxifen	vehicle	148.85 ± 5.95	14
Avil^{wt};*Cnr1*$^{lox/lox}$	tamoxifen	JD5037	150.64 ±14.25	15
Avil^{CreERT};*Cnr1*$^{lox/lox}$	tamoxifen	vehicle	179.74 ±21.83	11
Avil^{CreERT};*Cnr1*$^{lox/lox}$	tamoxifen	JD5037	143.27 ± 9.42	19
Avil^{wt};*Cnr1*$^{lox/lox}$	tamoxifen	vehicle	157.95 ±11.04	9
Avil^{wt};*Cnr1*$^{lox/lox}$	tamoxifen	PF-05190457	152.06 ±10.01	10
Avil^{CreERT};*Cnr1*$^{lox/lox}$	tamoxifen	vehicle	158.30 ±12.72	10
Avil^{CreERT};*Cnr1*$^{lox/lox}$	tamoxifen	PF-05190457	154.64 ±14.60	11
Avil^{wt};*Cnr1*$^{lox/lox}$	tamoxifen	vehicle	160.84 ± 9.37	8
Avil^{wt};*Cnr1*$^{lox/lox}$	tamoxifen	*S*-MRI1891	158.01 ± 9.79	6
Avil^{CreERT};*Cnr1*$^{lox/lox}$	tamoxifen	vehicle	162.68 ± 5.08	12
Avil^{CreERT};*Cnr1*$^{lox/lox}$	tamoxifen	*S*-MRI1891	160.75 ± 4.60	12
Trpv1^{wt};*Cnr1*$^{lox/lox}$		vehicle	207.39 ± 6.08	12
Trpv1^{wt};*Cnr1*$^{lox/lox}$		JD5037	187.44 ± 4.30	16
Trpv1^{Cre};*Cnr1*$^{lox/lox}$		vehicle	198.02 ± 5.12	21
Trpv1^{Cre};*Cnr1*$^{lox/lox}$		JD5037	174.09 ± 2.66	23

C57BL/6J		vehicle	187.79 ±48.92	10
C57BL/6J		*S*-MRI1891 1 mg/kg	130.06 ± 6.30	10
C57BL/6J		*S*-MRI1891 3 mg/kg	138.48 ± 5.95	10
C57BL/6J		*R*-MRI1891 3 mg/kg	135.01 ± 7.64	7
C57BL/6J	blank saporin	vehicle	145.51 ±17.48	6
C57BL/6J	blank saporin	JD5037	128.61 ± 3.23	6
C57BL/6J	CB1 saporin	vehicle	125.94 ± 2.07	6
C57BL/6J	CB1 saporin	JD5037	149.24 ±15.64	6
$Phox2b^{wt};Cnr1^{lox/lox}$		vehicle	134.52 ±11.21	11
$Phox2b^{wt};Cnr1^{lox/lox}$		JD5037	122.36 ±11.86	11
$Phox2b^{Cre};Cnr1^{lox/lox}$		vehicle	128.83 ± 4.44	13
$Phox2b^{Cre};Cnr1^{lox/lox}$		JD5037	113.77 ± 6.05	14
$Phox2b^{wt};Cnr1^{lox/lox}$		vehicle	153.74 ±23.47	8
$Phox2b^{wt};Cnr1^{lox/lox}$		*S*-MRI1891	156.71 ±25.48	8
$Phox2b^{Cre};Cnr1^{lox/lox}$		vehicle	140.64 ± 4.04	11
$Phox2b^{Cre};Cnr1^{lox/lox}$		*S*-MRI1891	166.05 ±16.18	12
$Phox2b^{wt};Cnr1^{lox/lox}$		vehicle	166.52 ±11.72	12
$Phox2b^{wt};Cnr1^{lox/lox}$		PF-05190457	170.56 ±24.12	10
$Phox2b^{Cre};Cnr1^{lox/lox}$		vehicle	203.76 ±22.61	10
$Phox2b^{Cre};Cnr1^{lox/lox}$		PF-05190457	195.71 ±23.40	10
$Wnt1^{wt};Cnr1^{lox/lox}$		vehicle	160.29 ±10.59	12
$Wnt1^{wt};Cnr1^{lox/lox}$		JD5037	142.44 ± 8.57	12
$Wnt1^{Cre};Cnr1^{lox/lox}$		vehicle	137.82 ± 4.45	14
$Wnt1^{Cre};Cnr1^{lox/lox}$		JD5037	165.16 ±11.66	15
$Glp1r^{wt};Cnr1^{lox/lox}$		vehicle	149.48 ± 6.37	10
$Glp1r^{wt};Cnr1^{lox/lox}$		JD5037	150.31 ± 7.53	10

Gpr65^{wt};*Cnr1*$^{lox/lox}$	13.52 ± 0.69	135.16 ± 3.77	28
Gpr65^{iresCre};*Cnr1*$^{lox/lox}$	12.90 ± 1.14	129.35 ± 4.64	22
Phox2b^{wt};*Ghsr*$^{lox/lox}$	12.06 ± 0.39 *	126.30 ± 2.91	51
Phox2b^{Cre};*Ghsr*$^{lox/lox}$	10.53 ± 0.46	121.70 ± 2.71	49

Values are means ± SEM from the indicated number (N) of experiments. Where appropriate, the Student’s t-test for unpaired samples, Welch's t-test or Mann Whitney U test was used. *$P < 0.05$, ***$P < 0.001$ by the Mann Whitney U test or by the Kruskal–Wallis test. A detailed description of the statistical tests used in the study can be found in the Materials and Methods section. Raw data points and the associated estimates of variation are accessible in the source data file.
